# *Gen*^*Htr*^: a tool for comparative assessment of genetic heterogeneity in microbial genomes generated by massive short-read sequencing

**DOI:** 10.1186/1471-2105-11-508

**Published:** 2010-10-12

**Authors:** GongXin Yu

**Affiliations:** 1 Department of Biological Science, Department of Computer Science, Boise State University, 1910 University Drive, Boise, Idaho 83725, USA

## Abstract

**Background:**

Microevolution is the study of short-term changes of alleles within a population and their effects on the phenotype of organisms. The result of the below-species-level evolution is heterogeneity, where populations consist of subpopulations with a large number of structural variations. Heterogeneity analysis is thus essential to our understanding of how selective and neutral forces shape bacterial populations over a short period of time. The Solexa Genome Analyzer, a next-generation sequencing platform, allows millions of short sequencing reads to be obtained with great accuracy, allowing for the ability to study the dynamics of the bacterial population at the whole genome level. The tool referred to as ***Gen^Htr ^***was developed for genome-wide heterogeneity analysis.

**Results:**

For particular bacterial strains, ***Gen^Htr ^***relies on a set of Solexa short reads on given bacteria pathogens and their isogenic reference genome to identify heterogeneity sites, the chromosomal positions with multiple variants of genes in the bacterial population, and variations that occur in large gene families. ***Gen^Htr ^***accomplishes this by building and comparatively analyzing genome-wide heterogeneity genotypes for both the newly sequenced genomes (using massive short-read sequencing) and their isogenic reference (using simulated data). As proof of the concept, this approach was applied to SRX007711, the Solexa sequencing data for a newly sequenced *Staphylococcus aureus *subsp. USA300 cell line, and demonstrated that it could predict such multiple variants. They include multiple variants of genes critical in pathogenesis, e.g. genes encoding a LysR family transcriptional regulator, 23 S ribosomal RNA, and DNA mismatch repair protein MutS. The heterogeneity results in non-synonymous and nonsense mutations, leading to truncated proteins for both LysR and MutS.

**Conclusion:**

***Gen^Htr ^***was developed for genome-wide heterogeneity analysis. Although it is much more time-consuming when compared to Maq, a popular tool for SNP analysis, ***Gen^Htr ^***is able to predict potential multiple variants that pre-exist in the bacterial population as well as SNPs that occur in the highly duplicated gene families. It is expected that, with the proper experimental design, this analysis can improve our understanding of the molecular mechanism underlying the dynamics and the evolution of drug-resistant bacterial pathogens.

## Background

Microevolution is defined as any evolutionary changes below the species level. It is the study of short-term changes within a population or a species of its alleles (alternative genes) and their effects on the phenotype of organisms that make up that population. The result of the below-species-level evolution is heterogeneity, where populations are made up of subpopulations with a large number of structural variations. Heterogeneity analysis is, therefore, essential to our understanding of how the selective and neutral forces shape bacterial populations over a short period of time [1-4].

In *S. aureus*, the role of microevolution is especially significant, in which heterogeneity is hypothesized to be the basic molecular mechanism for drug resistance [5]. Indeed, pre-existing drug-resistant mutants were often detected, from which drug-resistant mutants emerged during drug therapy or on the drug-containing growth medium. *S. aureus *strain of PC-1, PC-2 and PC-3 are great examples [6]. They were isolated at different stages of vancomycin therapy. The first two were recovered from a patient in the early stages of vancomycin therapy, whereas the third was isolated one day before the patient's death. The minimal inhibitory concentration of vancomycin is 2 μg per milliliter for PC-1 and PC-2, while PC-3 is capable of growing on agar plates containing vancomycin at a concentration of 8 μg per milliliter at a frequency of approximately 10^-3^. PC-3* is a colony picked from PC-3 strains capable of growing on agar containing 8 μg of vancomycin per milliliter. The new isolate can even grow on 16 μg of vancomycin per milliliter at a frequency of approximately 10^-5^, a strong indicator for the existence of subpopulations. Similarly, mutants with higher vancomycin resistance were also picked from MM66 plated on vancomycin containing agar with a minimum inhibitory concentration [7,8].

The heterogeneity is not unique to *S. aureus*. It is found in the population of *Mycobacterium tuberculosis *and many other bacterial pathogens, where the pathogen can gain drug resistance under the selective pressure of drug therapy. Post et al identified drug-resistant subpopulations within TB patients [9], in which four of the 13 patients acquired additional drug-resistance mutations during the course of treatment [9]. On the other hand, the bacterial pathogen could also lose its pathogenicity when selective pressure is absent, leading to the in vitro accumulation of attenuated mutants. A systematic analysis of individual clones isolated from subcultured *M. tuberculosis *H37Rv and a non-subcultured frozen stock detected a mixed population in H37Rv containing wild-type cells as well as neutral red and Phthiocerol Dimycocerosate (PDIM) mutants [10]. Microarray analysis confirmed a significant heterogeneity [10]. Heterogeneity was also found in natural bacterial populations of *Escherichia coli *[11], *Salmonella enterica *[12], *Neisseria meningitidis *[13], *Haemophilus influenzae *[14], *Helicobacter pylori *[15], *Streptococcus pneumoniae *[16] and *Pseudomonas aeruginosa *[17]. From these bacterial pathogens, hypermutation and the pre-existence of drug-resistant subpopulations were detected. It is thus logical to conjecture that the heterogeneity in bacterial populations is fundamental to the development of drug-resistant strains in these and possibly other bacterial species. Analysis tools that possess the ability to study heterogeneous bacterial populations and the dynamic of genetic changes in the populations would greatly improve our understanding of the molecular mechanisms of microevolution.

Molecular epidemiological analysis tools play critical roles in our current understanding of bacterial microevolution. The tools include IS6110-based restriction fragment length polymorphisms (RFLP); spoligotyping and the mycobacterial interspersed repetitive-unit-variable-number tandem-repeat (MIRU-VNTR) typing in the study of *M. tuberculosis *populations; multilocus sequence typing (MLST); amplified fragment length polymorphism analysis (AFLP); double-locus sequence typing (DLST); and spa typing in the analysis of *S. aureus population*. All of these techniques, however, have severe limitations. First, these tools focus on small sets of genome components, such as DNA sequences of internal fragments of multiple (usually seven) housekeeping genes in MLST [18], single-strand sequencing of partial repeat sequences of genes clfB and sp in DLST [19], and DNA sequence analysis of variable repeat regions of the protein A gene in spa typing [20]. Consequently, either the genetic heterogeneity could not be detected at all or its frequency is seriously underestimated [21-23].

The Solexa Genome Analyzer is a sequencing system powered by the next-generation Solexa sequencing technology [24]. It is based on massively parallel, shotgun, clonal sequencing-by-synthesis and is characterized by high throughput and precision in base calls. With this platform, millions of short reads can be obtained with an accuracy of up to 99% [25], allowing massively parallel picoliter-scale amplification and sequence determination of individual DNA molecules [26]. For example, a mixture of three HIV-1 envelope variants pooled in proportions of 89%, 10%, and 1% can be accurately detected [27]. Recently, Wang et al. detected an average of 58 variants per clinical HIV plasma samples using this technology compared to an average of eight variants per sample using conventional direct-PCR dideoxynucleotide sequencing [28]. It can be hypothesized that, with these technologies, bacterial genomes in a heterogeneous population can be adequately sequenced so that population dynamics in respect to genetic heterogeneity can be studied at the whole genome level.

Heterogeneity analyses are straightforward due to the small size of the viral genome and the rarity of sequence duplications in the genome. However, the heterogeneity analysis is expected to face a significant challenge in bacterial genomes. Sequence duplications, insertions and deletions are ubiquitous features of bacterial genomes, which resulted in gene families and super-families with many paralogs [29-32]. The resulting complexity would make it especially difficult to determine whether particular heterogeneity sites are due to mutations that occur between the paralogs or spontaneous mutations that generate heterogeneous bacterial populations.

A few computational tools have been developed for genome-wide variant analysis, including BFAST [33], RMAP [34] and Maq [35]. This software facilitates the fast and accurate mapping of short reads to detect sequencing errors, single nucleotide polymorphisms (SNPs) and indels with well-defined statistics. For example, Maq calls the consensus based on a statistical model that maximizes the posterior probability [35]. All these tools, however, have limited or no capability for genetic heterogeneity analysis. Even though heterozygotes are called in the Maq analysis process, all analyses are local in nature, meaning that the process narrows down particular genome positions without considering global genome contexts. As a consequence, it is difficult to determine whether the heterogeneity is due to intra-genome variation or to the heterogeneous population. In addition, like many other tools, Maq has limited capability to reveal mutations that occur in repetitive DNA. As a matter of fact, SNPs lying in repetition were intentionally excluded from analysis [36].

Here, a new tool referred to as ***Gen^Htr ^***was established. ***Gen^Htr ^***is unique in that it could detect variants within the context of whole genomes. Specifically, the software first establishes the genotype in genetic heterogeneity at all chromosomal positions for both newly sequenced bacterial strains (based on the Solexa reads) and their isogenic reference genomes (based on the simulated data). Comparative genotype analysis allows for the identification of heterogeneity sites where the chromosomal positions are heterogeneous in newly sequenced genomes but homogenous in the isogenic reference genome. Together with Maq, the software then provides clues to determine whether the multiple variants are due to intra-genome duplications, sequencing artifacts or spontaneous mutations, thus helping to prioritize the variants for experimental validation. In addition, GeneWise [37,38] was integrated so that synonymous/non-synonymous mutations can be analyzed as well.

To prove this concept, I applied this approach to SRX007711, the Solexa sequencing data downloaded from NCBI Sequence Read Archive (SRA) for a newly sequenced *S. aureus *subsp. USA300 cell line. The data set was chosen as a user model organism for the following reasons: First, completely sequenced genomes of two USA300 strains are available in the NCBI [Genome Assembly/Annotation Projects]; second, *S. aureus *is one of the leading causes of infectious disease mortality; and third, heterogeneity has been implicated in the process of drug-resistance development where *S. aureus *undergoes genetic shifts during treatment, resulting in the acquisition of subtle genetic changes in *S. aureus *subpopulations [39]. It is imperative to find cures effective in treating *S. aureus *infections since failures can lead to a dire consequence: the selection and spread of multiple drug-resistant strains [40]. This paper illustrates the analysis procedure and presents partial results.

## Methods

### Genome sequence data

The Solexa genome sequences (Raw Solexa sequence reads) of *S. aureus *strain were downloaded from ftp://ftp.ncbi.nlm.nih.gov/sra/static/SRX007/SRX007710/. The *S. aureus *is a USA300 strain that was sequenced using Illumina sequencing technology by the BROAD Institute in the Staphylococcus_aureus_Assembly_Development project. In addition, the completely sequenced *S. aureus *genome of *S. aureus *USA300 FPR3757 was downloaded from NCBI ftp://ftp.ncbi.nih.gov/genomes/Bacteria/Staphylococcus_aureus_USA300_FPR3757/. Like the newly sequenced strain, this strain belongs to the aureus USA300 subspecies. Its genome includes a chromosome of 2872769 base pairs (bps) (NC_007793) and three plasmids of 3125 bps (NC_007790), 4439 bps (NC_007791), and 37136 (NC_007792), respectively. The USA300 subspecies is methicillin-resistant, community-acquired (CA-MRSA) and has been involved in epidemiologically unassociated outbreaks of skin and soft tissue infections in healthy individuals in at least 21 U.S. states, as well as in Canada and Europe [41].

### *Gen^Htr ^*Steps

***Gen^Htr ^***is based on a seamless integration of various computational tools such as MegaBlast, Blat and GeneWise as well as some in-house-developed Perl modules. MegaBlast uses a greedy algorithm for the nucleotide sequence-alignment search, up to 10 times faster than more common sequence-similarity programs [42]. Blat is a BLAST-Like Alignment Tool, designed specifically for accurate and faster sequence alignments [43]. For the heterogeneity analysis of a particular bacterial strain, a completely sequenced genome of an isogenic strain is selected as a reference genome, named IRG for short.

**Figure 1:**
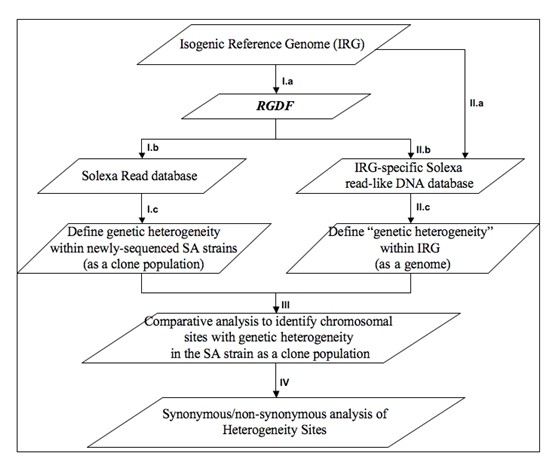
**The four-step analysis procedure for heterogeneity analysis of bacterial population**. The first step is to establish genome-wide heterogeneity phenotypes for the newly sequenced bacterial strain (a clone population) (**I**.). The step first creates a database of *R*eference *G*enome *D*NA *F*ragments (*RGDF*) with a set of non-overlapped DNA fragments from the isogenic reference genome (IRG) (**I.a**); Once established, *RGDF *is used to search the database of Solexa short reads of the bacterial strain via MegaBlast to identify its candidate trace sequences (**I.b**), which are then mapped to the IRG to define genetic heterogeneity (**I.c**). The second step is a simulation procedure to study the genome complexity of the IRG (**II**.). The procedure creates an IRG-specific DNA database, covering all possible *N*-base pair "Solexa read-like" DNA fragments from the IRG (**II.a**), Then the analysis follow the same procedure from **I.b **to **I.c **to identify candidate sequences from the IRG-specific DNA database (**II.b**) and to establish genome-wide "heterogeneity" genotypes (**II.c**). The genotypes from step I and II are comparatively analyzed (**III**.). The genetic heterogeneity sites were analyzed with genewisedb for synonymous/non-synonymous mutations (**IV**).

***Gen^Htr ^***is partitioned into four conceptual steps (Fig. 1). The first step is to create the database ***R***eference ***G***enome ***D***NA ***F***ragments (***RGDF***), an entire set of non-overlapped DNA fragments from the IRG (**Fig.1 I.a**). The ***RGDF***s have a pre-defined length, e.g. a default length of 1,000 base pairs, which was empirically determined. Larger ***RGDF***s often present a significant challenge since Solexa creates massive sequencing reads in certain genomic areas that often overwhelm MegaBlast, a key tool in this analysis. In such case, no sequence alignments will be displayed.

Once established, ***RGDF ***is used to search genome-specific databases of Solexa short reads via MegaBlast to identify its candidate trace sequences (Fig.1 I.b). MegaBlast is run with all default parameters except -v and -b. Both are assigned a value of 1,000,000 to allow all possible database sequences to show one-line descriptions (-v) and alignments (-b). The candidate trace sequences were defined as those that display at least 95% sequence identity with a given ***RGDF***. The 95% is used to limit alignments between Solexa Reads and ***RGDF***s to those of at most two mismatches.

Candidate trace sequences are then mapped to the IRG based on the alignments from the MegaBlast analysis (Fig.1 I.c) to define genetic heterogeneity within a newly sequenced bacterial strain (treated as a population). In the mapping, nucleotide identities (**D_ij_**) are determine at each chromosomal position, where **D_ij _**represents the **j^th ^**alternative nucleotide at chromosomal position **i**. **D_ij _**is identical either to those in the reference genome or to those that are substituted, deleted or inserted. Furthermore, the numbers of candidate Trace sequences (**M_ij_**) used to derive nucleotide identity **j **at position ***i ***as well as the percentage sequence identities **(P_ij_) **between the candidate trace sequences and the ***RGDF ***are recorded. As a result, the genetic heterogeneity genotype in the format of **P_i1_:D_i1_:M_i1_, P_i2_:D_i2_:M_i2 _... P_ii_:D_ij_:M_ij _... P_in_:D_in_:M_ik _**will be established for all chromosomal positions. Moreover, a 2-2-5 rule is designed specifying that any chromosomal position will be defined as candidate genetic heterogeneity sites if the following conditions are satisfied: First, there must exist at least two alternative nucleotides at certain positions; and second, among the alternative nucleotides at these positions, there must be at least five Solexa reads per an alternative with an average Phred value of 13 (a probability of *p *that is no greater than 0.05 to be incorrect base calls) [26]. At least five reads were empirically required for accurate single nucleotide mutation calling [44].

The second step is a simulation procedure to analyze genome complexities of DNA mixtures derived from the isogenic reference genome. The procedure first creates an IRG-specific DNA database, where all possible ***N***-base pair "Solexa read-like" DNA fragments are generated from the IRG (Fig.1 II.a). A moving window of ***N***-base pairs is used to scan over the genome to generate DNA fragments. The length of Solexa reads determines the size of the moving window (***N = ***37 base pairs in this analysis). Then the analysis follows the exact procedures from Fig.1 I.b and I.c to identify candidate sequences from the IRG-specific DNA database (Fig.1 II.b) and establish genome-wide "heterogeneity" genotypes (Fig.1 II.c). For consistency, the term "heterogeneity" is still used in this section, but instead of describing the heterogeneous bacterial population, it is used to portray the genome complexity of the IRG. The read depth from simulated data varies from genomic area to genomic area, depending on the complexity of the DNA. In the areas of single copy, the read depth is equal to ***N***, the width of the moving window.

The genotypes from this and the previous step are compared (Fig.1 III.) to identify the genetic heterogeneity sites where alternative nucleotides were found from the analysis of Solexa sequence data but not from the analysis of the reference genome. Once the genetic heterogeneity sites were predicted, synonymous/non-synonymous mutations at the sites were analyzed with genewisedb (GeneWise) [37,38] (Fig.1, IV.), where candidate trace sequences with heterogeneity sites were comparatively analyzed with their orthologous proteins from the isogenic reference genome. The genewisedb is run with all default parameters.

***Gen^Htr ^***runs are semi-automated. A run of ***Gen^Htr ^***on the 700 M base pairs of *S. aureus *takes ~ 10 h on a 500 MB RAM Pentium 4 computer running Linux. Running time increases linearly with the size of the Solexa genome sequences and genome length.

## Results and discussion

### Characterization of the model genome

In heterogeneity analyses, a key pre-requirement is the existence of completely sequenced, assembled genomes of an isogenic bacterial strain. In NCBI, many completely sequenced *S. aureus *genomes are available, including those for FPR3757 and TCH1516, two strains of *S. aureus *subspecies USA300. The data set SRX007711, a USA300 strain, was selected as the model organism. The SRX007711 comes from the same subspecies. It was assumed that the newly sequenced genome was highly similar to the two sequenced subspecies. Indeed, using *S. aureus *subspecies USA300 FPR3757 as a template, ***Gen^Htr ^***can reconstruct the entire genome of SRX007711 with merely five gaps to a total of 410 base pairs (Additional file 1** Table S1**). Moreover, only 101 substitutions, 2 insertions and 3 deletions were revealed, suggesting that SRX007711 is isogenic to FPR3757. Another critical pre-requirement is the size of the sequencing data. This analysis indicated that SRX007711 has massive coverage of the bacterial genome with an average number of up to 130 Solexa reads per mapped chromosomal position. Among these chromosomal positions, about 77.37% have greater than 100 of Solexa reads, with a maximum of 1,346.

### Analysis of the isogenic reference genome

The results indicated that the simulation in ***Gen^Htr ^***could characterize genome complexity of the IRG. First, it determined whether chromosomal positions occur in single-copy-DNA fragments or in duplicated DNA fragments (Additional file 2** Table S2**). The analysis also revealed "heterogeneity" within the IRG, a mixture of DNA fragments derived from recent duplication and subsequent mutations. A total of 981 "heterogeneity sites" were detected at genic areas (Additional file 3** Table S3**). A majority of the "heterogeneity sites" are present in proteins important to pathogenesis (about 84% if genes encoding phage-like, transposase and hypothetic proteins were excluded). These include 5 sites on the gene for the immunoglobulin G binding protein A precursor, 8 sites on the gene for the superantigen-like protein, 12 sites on the gene for the cell surface proteins, 18 sites on the gene of teichoic acid biosynthesis protein, 36 sites on the gene of fibronectin binding protein A/B, 71 sites on the genes of clumping factor A/B, 191 sites on the genes of sdrC/D/E proteins and 135 sites on the genes of staphylococcal tandem lipoprotein. Many of those genes encode surface proteins that have a common C-terminal LPXTG/NPQTN cell wall attachment motif, a sequence fragment that plays an essential role in host colonization, biofilm formation, and the evasion of host defense [45]. For example, wall teichoic acids (WTA) have been shown to be essential for the survival of *Bacillus subtilis *[46] and function as virulence factors in *S. aureus *[47,48]. Deficient WTA-mutants were impaired in their adherence to nasal cells and were unable to colonize cotton rat nares [47,49]. The superantigen-like protein is a bacterial protein toxin that binds to the major histocompatibility complex class II and T-cell receptor to stimulate large numbers of T cells, leading to toxic shock syndrome [50].

The detection of extensive "heterogeneity" from the isogenic reference genome, especially on the pathogenesis-related genes, is not surprising. Gene duplication has long been recognized as one of the most important mechanisms in the evolution of bacterial genomes, creating multiple homologs within a genome. The paralogous gene groups are further involved in mutations and genome rearrangements that help the bacteria adapt to ever-changing environments [51]. By "heterogeneity" analysis, ***Gen^Htr ^***can characterize the genome dynamics of bacterial pathogens.

### Heterogeneity analysis of the newly sequenced *S. aureus *subsp. USA300 cell line

The genetic heterogeneity analysis of SRX007711, the Solexa data from the newly sequenced USA300 cell line, detected a similar phenomenon. A total of 2,056 heterogeneity sites were identified through heterogeneity analysis. Among them, 204 are unique to the new cell line when compared to the "heterogeneity" genotypes of its isogenic reference genome. Many of them were also detected by Maq as single nucleotide polymorphisms (SNPs) and passed the "SNPfilter" when Solexa reads that cover the heterogeneity sites were extracted and used as an alternative Maq input (**Section I in **Table 1). The heterogeneity site that was detected at the gene coding for sensor histidine kinase is a good example. Among the 123 candidate trace sequences mapped to this site, 115 have a nucleotide that is identical to the isogenic reference genome and 7 with a G- > C substitution. All seven substitutions have a Phred value greater than or equal to 27 in the base calling and three of them have a Phred value of 40, the highest possible quality value in the data set. A value of 40 has a converted probability of 0.0001 incorrect reads. In contrast, the position is homogeneous in the isogenic reference genome. The same phenomenon was observed at other heterogeneity sites, including those at the genes encoding for the sulfatase family protein, the lipoate-protein ligase A family protein, the penicillin-binding protein 3, the lantibiotic epidermin biosynthesis protein EpiC, the oxacillin resistance-related FmtC protein, and the putative fibronectin/fibrinogen binding protein. Furthermore, a majority of the heterogeneity sites are located in the single-copy DNA fragment in the isogenic reference genome.

**Table 1:** Characterization of the genetic heterogeneity Sites and SNP in large gene families

Maq	Chrom Position	Genotype profile at selected loci of SRX007711	Genotype profile at selected loci of FPR3757	Read Depth	SRX007711 Mean Phred Values	Max Phred Value	Functional Description
	I. Heterogeneity sites that passed the SNPfilter and have an average of per-base Phred value greater than 13						

*	778416	T:6 G:140	G:37	6	31.5	40	sulfatase family protein
*	2512836	C:7 G:115	G:37	8	31.25	40	sensor histidine kinase
*	107624	A:5 C:110	C:37	5	29.8	40	hypothetical protein
*	435033	T:5 G:98	G:37	5	28.2	38	hypothetical protein
*	1021087	T:5 G:151	G:37	5	26.2	40	lipoate-protein ligase A family protein
*	1662849	A:5 C:134	C:37	6	24.66	40	penicillin-binding protein 3
*	2648343	A:5 C:192	C:37	5	24.6	40	drug transporter phosphotransferase system, glucose-
*	2674216	A:5 C:170	C:37	5	24.6	40	specific IIABC component
*	1542366	T:6 G:129	G:37	6	24.5	40	hypothetical protein lantibiotic epidermin biosynthesis
*	1950547	A:5 C:164	C:37	5	23.4	40	protein EpiC
*	105211	A:6 G:132	G:37	8	20.3	40	hypothetical protein
*	1857182	A:14 G:85	G:37	14	19.42	40	hypothetical protein phiSLT ORF2067-like protein, phage
*	1558524	T:5 G:56	G:37	7	17.57	40	tail tape measure protein phi77 ORF014-like protein, phage anti-
*	2122182	C:122 G:6	C:37	6	17.28	38	repressor protein
*	1383603	A:5 G:128	G:37	5	15.8	20	oxacillin resistance-related FmtC protein
		(A:2) T:5					lactose phosphotransferase system
*	2333470	C:164	C:37	7	15.28	40	repressor
							putative fibronectin/fibrinogen binding
*	1206348	A:1 T:6 G:145	G:37	7	14.71	25	protein
		A:153 (T:1)					
*	2262790	G:6	A:37	7	13.14	29	cation efflux family protein

	II. Heterogeneity sites that did not pass the SNPfilter but have an average per base Phred value greater than 13						

	1180638	T:5 G:97	G:37	5	40	40	cell division protein ftsA
							2-oxoglutarate dehydrogenase E1
	1437922	C:149 G:5	C:37	5	39.2	40	component
	2212436	A:5 C:107	C:37	5	37.8	40	thiamine-phosphate pyrophosphorylase
	257712	T:5 G:101	G:37	5	35.6	40	sensor histidine kinase family protein
	861340	A:9 C:136	C:49	10	34	40	clumping factor A
							acetyl-CoA carboxylase, biotin carboxyl
	1714319	T:6 C:95	C:37	6	34	40	carrier protein
	1252956	T:9 G:125	G:37	9	33	40	DNA topoisomerase I
							lantibiotic epidermin leader peptide
	1948255	A:5 C:169	C:37	5	32.8	40	processing serine protease EpiP
	955972	A:5 C:145	C:37	5	32.6	40	Hypothetical protein
	2123183	A:25 X:29	A:37	29	31.44	40	putative phage transcriptional regulator
	2638027	A:5 C:112	C:36	5	31	40	gluconate kinase
	1829558	T:5 G:118	G:37	5	30.8	40	septation ring formation regulator EzrA
							putative maltose ABC transporter,
	247386	A:5 C:163	C:37	5	30.4	40	maltose-binding protein
	2262622	C:5 G:156	G:37	5	30.4	40	cation efflux family protein
	344352	C:460 G:7	C:131	8	29.5	40	Hypothetical protein
	346978	C:383 G:7	C:113	8	29.5	40	Hypothetical protein
	472492	T:5 G:194	G:37	5	29.4	40	Hypothetical protein
	2175831	A:585 G:8	A:229	9	28	40	5 S ribosomal RNA
	1753468	C:96 G:5	C:37	5	27.2	40	Hypothetical protein
	1503664	A:113 T:6	A:37	6	27.16	40	Hypothetical protein
	617974	A:374 T:5	A:99	5	26.2	38	sdrD protein
	154794	A:5 C:136	C:37	5	25.6	40	Fe/Mn family superoxide dismutase
	1943616	C:5 G:110	G:37	5	25.2	32	serine protease SplA
	2064321	C:113 G:6	C:37	6	24.8	40	Hypothetical protein
	910508	A:177 T:6	A:37	7	24	40	lipoyl synthase
							5-methyltetrahydropteroyltriglutamate--
	408863	T:6 G:160	G:37	6	23.16	40	homocysteine S-methyltransferase
	2417570	A:5 G:109	G:37	5	22.8	39	Na+/H+ antiporter NhaC
							glycerol uptake operon antiterminator
	1311574	A:5 C:181	C:37	5	22.6	40	regulatory protein
							capsular polysaccharide biosynthesis
	175115	X:6 G:136	G:37	6	21.33	40	protein Cap5B
	2775087	A:14 T:107	T:49	14	20.4	29	clumping factor B
	2678195	A:5 G:92	G:37	5	19.4	27	LysR family transcriptional regulator
	451366	C:5 G:119	G:37	5	19.2	33	Superantigen-like protein 5
	1633215	A:5 C:83	C:37	5	18.4	28	putative traG membrane protein
	2114835	A:6 C:136	C:37	7	17.14	27	phiPVL ORF046-like protein
							
	1859648	C:5 X:1 G:121	G:37	6	16	24	FtsK/SpoIIIE family protein
	467549	A:89 T:23	A:58	23	15.56	40	Staphylococcal tandem lipoprotein
	2123177	A:57 X:5	A:37	5	15.4	21	putative phage transcriptional regulator
	36501	A:29 G:391	G:192	29	15.13	40	putative transposase
	1857109	A:162 G:12	A:37	12	14	40	hypothetical protein
	950365	A:5 C:113	C:37	5	13.8	17	Exonuclease RexB
	801123	T:5 G:91	G:37	5	13.4	28	transferrin receptor
	2481059	T:5 G:148	G:37	5	13.4	21	response regulator protein
	1545118	A:15 T:109	T:37	15	13.2	40	putative lipoprotein

	IV. Heterogeneity sites at RNA genes that pass the SNPfileter when single RNA genes were used as reference sequence.						

*	1997102	A:69 T:13	A:37	13	7.0	30	Leu tRNA
	1996261	T:7 C:76	C:55	8	14.25	30	Met tRNA
*	1961354	T:12 C:25	C:55	12	20.16	40	Met tRNA
	517898	T:581 C:8	T:231	8	31.37	40	5 S ribosomal RNA
*	556291	T:560 C:16	T:225	16	20.56	40	5 S ribosomal RNA
	561501	T:580 C:8	T:229	8	31.37	40	5 S ribosomal RNA
	1997607	A:569 G:8	A:218	8	31.37	40	5 S ribosomal RNA
	2292385	A:549 G:8	A:218	8	31.37	40	5 S ribosomal RNA
	516288	A:34 G:408	G:185	34	14.11	40	23 S ribosomal RNA
	517172	T:6 G:495	G:185	6	13.83	26	23 S ribosomal RNA
	559891	A:34 G:408	G:185	34	14.1	40	23 S ribosomal RNA
	560775	T:6 G:495	G:185	6	13.83	26	23 S ribosomal RNA
	1998333	A:6 C:495	C:185	6	13.8	26	23 S ribosomal RNA
	1999217	T:34 C:408	C:185	34	14.11	40	23 S ribosomal RNA
	2176557	A:6 C:495	C:185	6	13.83	26	23 S ribosomal RNA
	2177441	T:34 C:408	C:185	34	14.1	40	23 S ribosomal RNA
	2293111	A:6 C:495	C:185	6	13.83	26	23 S ribosomal RNA

### Heterogeneity analysis of the genes of single DNA copies

Further analysis indicated that the genetic heterogeneity has certain unique properties. First, a majority of the genetic heterogeneity sites were detected in unique genomic areas. Many of them have non-synonymous mutations, leading to amino acid alterations in target proteins (Table 2). For example, the genetic heterogeneity leads to a Q^59^- > K^59 ^substitution on putative fibronectin/fibrinogen binding protein, a C^717^- > Y^717 ^on oxacillin resistance-related FmtC protein at a ratio of 3.87% [T:6 G:145], and 3.7% [A:5 G:128] out of the detected candidate sequences, respectively. The genetic heterogeneity also leads to truncated proteins, e.g. at genes encoding sensor histidine kinase at a percentage of 5.74% [C:7 G:115], and phosphotransferase system, glucose-specific IIABC component at a percentage of 2.85% [A:5 C:170].

**Table 2:** Synonymous and non-synonymous analysis of mutations in the heterogeneity sites

Chrom Position	Types of Mutations	Genotype at SRX007711	Genotype at FPR3757	Alignment with Orthologous Proteins			Gene and Function
				gi 87161249 ref	423	EKDATIEKSNTG	
						E DATIEKSNTG	
						ENDATIEKSNTG	
				SRR022865_26088	1	gaggaagataag	
778416	NONSYN	T:6 G:140	G:37			aaacctaacacg	
						attattgaacat	sulfatase family protein lipoate-protein ligase A family protein

1021087				Query: 36 VKLAMEEYVLKN	1		
	NONSYN	T:5 G:151	G:37	+ LAMEEYVLKN			
				Sbjct: 14 LNLAMEEYVLKN	25		

				gi 87162345 ref	143	AGIGRYLLNRVD	
						AGIGRYLLNR+D	
						AGIGRYLLNRLD	
				SRR022865_32431	-37	Ggagattcaatg	lantibiotic epidermin biosynthesis protein EpiC
						Cgtggattagta	
1950547	NONSYN	A:5 C:164	C:37			Tgagatgataat	

				gi 87160275 ref	122	NCLPVYKILLEK	
						NCLPVYKILL+K	
						NCLPVYKILLKK	
				SRR022865_59666	-36	attcgtaattaa	Lactose phosphotransferase system repressor phosphotransferase system, glucose-specific IIABC component
						agtctaatttaa	
2333470	NONSYN	T:5 C:164	C:37			ttgggtatgaaa	

				Query: 36 LV*IAPWLKNDI	1		
				LV IAPWLKNDI			
2674216	TRUNC	A:5 C:170	C:37	Sbjct: 41 LVEIAPWLKNDI	52		

				gi 87161394 ref	71	KKVLLTGLGIVI	
						KK+LLTGLGIVI	
						KKLLLTGLGIVI	
				SRR022865_30969	1	aatctagtgaga	
2648343	NONSYN	A:5 C:192	C:37			aatttcgtgttt	drug transporter

						aaatagagaaac	
				gi 87160343 ref	120	EVQSKEMLIISI	
						EVQSKEMLI+SI	
						EVQSKEMLIVSI	
				SRR022865_47009	2	ggctagatagaa	
2262790	NONSYN	A:153 G:6	A:37			atacaattttgt	cation efflux family protein

						ataaaagacttt	
				gi 87160156 ref	825	SDSDSDSDSDSD	
						SDSDSDSDSDSD	
						SDSDSDSDSDSD	
				SRR022865_28556	-37	agtgtgagtgtg	
861340	SYN	A:9 C:136	C:49			gacacagacaca	clumping factor A
						ctatatctatac	

				gi 87160605 ref	4	FTQLSDRIKKAI	
						FTQLSDRIKK I	
						FTQLSDRIKKDI	
				SRR022865_49291	-36	tactagaaaaga	
1542366	NONSYN	A:14 G:85	G:37			tcatgagtaaat	hypothetical protein
						ttagttacaacc	

				gi 87160966 ref	476	RNDMVEFFGEKL	5-
						RN+MVEFFGEKL	
						RNEMVEFFGEKL	Methyltetrahydropteroyltriglut amate--homocysteine S- Methyltransferase
				SRR022865_53088	1	cagaggttggat	
						gaattattgaat	
408863	NONSYN	T:6 G:160	G:37			ttagtaccaaaa	

				gi 87161941 ref	177	VDVLDVYSDAY	
						VD+LDVYSDAY	
						VDLLDVYSDAY	
				SRR022865_54601	3	ggttggttggt	
1180638	NONSYN	T:5 G:97	G:37			tattatacaca	cell division protein ftsA
						ttaattcttat	

				gi 87160920 ref	104	KGDIIGYVEAMK	
						KGDIIGYVEA+K	
						KGDIIGYVEAIK	
				SRR022865_82913	-37	aggaagtgggaa	acetyl-CoA carboxylase, biotin carboxyl carrier protein
						agattgatacta	
1714319	NONSYN	T:6 C:95	C:37			gattaattagaa	

				gi 87162179 ref	415	AKSEVWRQMMSD	
						AKSEVWRQM+SD	
						AKSEVWRQMISD	
				SRR022865_51952	-36	gaaggtccaatg	
						cagatggattca	
2638027	NONSYN	A:5 C:112	C:36			gataagtagtat	gluconate kinase

				gi 87161981 ref	47	DIAVVDIMMDGM	
						DIAVVDIMMD M	
						DIAVVDIMMDVM	
				SRR022865_55616	-36	gaggggaaagga	
						atcttatttatt	
2481059	NONSYN	T:5 G:148	G:37			ttagattggttg	response regulator protein

				gi 87161886 ref	196	KSENIEKTVNRF	
						K ENIEKTVNRF	
						KIENIEKTVNRF	
				SRR022865_56405	1	aagaagaagact	
						ataataactagt	thiamine-phosphate pyrophosphorylase
2212436	NONSYN	A:5 C:107	C:37			atattagtttac	

				gi 87161325 ref	64	PVVKELKKHAK	
						P VKELKKHAK	
						PFVKELKKHAK	
				SRR022865_71442	3	ctgagtaacga	
						cttaataaaca	
1252956	NONSYN	T:9 G:125	G:37			ttaaagaataa	DNA topoisomerase I

				gi 87162241 ref	319	SMDNVVTVGSTD	
						SM NVVTVGSTD	
						SMYNVVTVGSTD	lantibiotic epidermin leader
				SRR022865_97728	2	tataggaggtag	
1948255	NONSYN	A:5 C:169	C:37			ctaattctgcca	peptide processing serine
						tgctctaaatat	protease EpiP

1309034	TRUNC	C:115 G:5	C:37	Query: 35 ASAGKKSSI*N	3 **^b^**		DNA mismatch repair protein MutS
				Sbjct: 754 ASAGKKSSISN	764		

				gi 87161934 ref	619	QQANVELSPTSD	
						Q+ANVELSPTSD	
						QKANVELSPTSD	
				SRR022865_10478	2	cagaggtacatg	
950365	NONSYN	A:5 C:113	C:37			aacatatgccca	
						ggttcgataaat	exonuclease RexB

				Query: 1 FK*YFKQFEENY	36		
				FK YFKQFEENY			
				Sbjct: 223 FKSYFKQFEENY	234		
2512836	TRUNC	C:7 G:115	G:37				sensor histidine kinase

				Query: 3 Q*IINDEVDIG	35		
				Q IINDEVDIG			LysR family transcriptional regulator
2678195	TRUNC	A:5 G:92	G:37	Sbjct: 137 QQIINDEVDIG	147		

Note. TRUNC: truncated protein; NONSYN: non-synonymous mutation and SYN: synonymous mutation. **^a^**: align with Genewisedb and **^b^**: align with Blastx.

The genetic heterogeneity is involved in some pathogenesis-related genes, including penicillin-binding proteins (PBP), fibrinogen/fibronectin-binding proteins (Fnbp), and lipoate-protein ligase A family protein. PBP is a key player in the bacterial cell cycle and drug-resistance processes. Altered PBPs with a reduced affinity to penicillin lead to penicillin resistance in clinical isolates of *S. pneumoniae *[52-54]. Fnbp is a major platelet-activating factor on the surface of S.aureus [55]. Truncated derivatives of the genes promote platelet activation when expressed on the surface of *S.aureus *or *Lactococcus lactis*, indicating two distinct mechanisms of activation. fmtC is a gene that affects oxacillin resistance in methicillin-resistant *S. aureus*. Its mutants showed increased susceptibility to beta-lactam antibiotics and bacitracin [56]. The gene of lipoate-protein ligase A family protein is upregulated by daptomycin [57]. The antibiotic induces the *S. aureus *cell wall stress stimulon and genes responsive to membrane depolarization.

As indicated above, Maq was used as an important tool for validation. However, many genetic heterogeneity sites could not pass the "SNPfilter" (**Section II in **Table 1), but it is worth illustrating them from the functional perspective because of their potential roles in the pathogenicity and microevolution of *S. aureus *strains. The sites include a LysR family transcriptional regulator gene with a genotype of [A:5 G:92]. A nonsense mutation results in a truncated LysR family transcriptional regulator. This protein is a global transcriptional regulator, acting as either activators or repressors of single or operonic genes. It regulates a diverse set of genes, including those involved in virulence, metabolism, quorum sensing and motility [58,59]. In mycobacteria, the lack of mismatch correction is recognized as a promoter of mycobacterial evolution [60]. It is, however, not completely clear how the genetic variation and phenotypic diversity are created in *S. aureus*. The discovery of the multi-variants in the genes that are known to be associated with hypermutability will shed light on the molecular mechanisms. The first such site is located at the gene encoding DNA mismatch repair protein MutS. The heterogeneity site has a genotype of [C:115 G:5]. The C- > G substitution at the chromosomal position of 1,309,034 results in a nonsense mutation, leading to a truncated protein with a loss of 77 amino acids at the C-terminal of the protein. The finding of truncated DNA mismatch repair protein MutS in the newly sequenced *S. aureus *subsp. USA300 cell line is new but not a surprise. This gene is part of the bacterial mismatch repair system. It functions to correct point mutations and small insertions/deletions that fail to be proofread by DNA polymerase activity. Previous experiments indicated that its disruption is related to the high-frequency hypermutability and drug-resistant mutants. In *P. aeruginosa*, mutS mutants displayed an increase in antibiotic-resistance. Furthermore, antibiotic-resistant levels of the generated mutants were higher than those measured from spontaneous resistant mutants derived from wild-type cells [61]. In *S. aureus*, the inactivation of MutS or MutL was associated with the emergence of a hypermutator phenotype that favors the acquisition of antibiotic resistance and facilitates bacterial adaptation during long-term persistence [62]. Drug-resistant subpopulations with mutant genes coding for DNA repair enzymes, referred to as strong mutators, were found in the laboratory populations of *E. coli *[63]; *S. enterica serovar Typhimurium *[12] and in natural populations of *E. coli *[11]; *S. enterica *[64]; *N. meningitidis *[13]; and *P. aeruginosa *[65]. All of them have a defective mismatch repair system due to the inactivation of mutS or mutL genes.

Additional candidates to establishing the genetic diversity necessary for the evolution of drug-resistant strains are the variants found at genes of exonuclease RexB and DNA topoisomerase I. The former has a genotype of [A:5 C:113] at the chromosomal position of 950,365, and the latter has a genotype of [T:9 G:125] at the chromosomal position of 1,252,956. Substitutions at both positions lead to non-synonymous mutations, causing amino acid replacements: Q^620^- > K^620 ^at exonuclease RexB protein and V^65^- > F^65 ^at topoisomerase I protein. The RexB is part of the cellular system that plays a crucial role in homologous recombination for the repair of a variety of DNA damage, involving in the maintenance of chromosome integrity and the generation of genetic variability [66]. Topoisomerases are essential enzymes that solve topological problems arising from the double-helical structure of DNA. It is, then, related to the status of DNA supercoiling, a critical factor that modulates the expression of virulence genes in pathogenic bacteria at different phases of the host-pathogen relationship [67]. Validation of the heterogeneity sites by targeted-sequencing with different technologies would improve our understanding of the molecular mechanisms underlying the evolution and pathogenicity of *S. aureus*.

### Detection of mutations in the large gene families

Mutations were not only detected in the unique genes but also in large gene families, e.g. 5 S rRNA and 23 S rRNA genes (**Section III in **table 1). The mutations in these gene families were further validated through Maq with high confidence, where individual sequences from the genes were used as references. The results are not surprising. Previous experiments established a clear association between mutations in the ribosomal genes and hypermutability. Canu et al. detected a variety of ribosomal mutations that conferred resistance to macrolides, clindamycin, streptogramin, and

telithromycin in *Streptococcus pneumoniae *[68]. Prunier et al. provided additional support for the role of the rRNA gene in drug resistance and hypermutability, where six strains of *S. aureus *were isolated from cystic fibrosis patients after treatment with azithromycin and all carried either A2058G/U or A2059G mutations within the 23 S rRNA gene and all acquired cross-resistances to azithromycin and erythromycin, the two therapeutic agents [69].

Mutations were also detected in the sequences that coded for clumping factor A/B and sdrD proteins. Clumping factor A/B and sdrD protein are pathogenic factors from two large gene families in the *S. aureus*. Clumping factor A (ClfA) is a fibrinogen-binding protein expressed on the *S. aureus *cell surface and has previously been shown to act as a virulence factor in experimental septic arthritis [70]. Clumping factor B (ClfB) is one of the microbial surface components that recognizes adhesive matrix molecules, mediates the adherence of S. aureus to immobilized fibrinogen and promotes bacterial clumping in soluble fibrinogen [71]. Just like clumping factor B, the serine-aspartic acid repeat protein SdrD can also promote adhesion to squamous cells [72]. A mutant strain that lacks SdrD proteins can cause defective in adherence [73].

### Advancement of the tool

The success of the demonstration indicated that ***Gen^Htr ^***is an improvement over available tools to calculate genetic heterogeneity. For instance, one of the biggest challenges in the Solexa-based heterogeneity analysis, specifically with Maq, is the low frequency of mutant subpopulation [6]. Using the new sequence data of the *S. aureus *USA300 cell line, Maq detected about 119 single nucleotide polymorphisms (SNPs) but missed a majority of the heterogeneity sites detected by the ***Gen^Htr ^***analysis. Improvement was made when Solexa reads that cover the heterogeneity sites were extracted and used as an alternative Maq input. Additional heterogeneity sites were indeed rediscovered but not all of them. This is because a greater part of the heterogeneity sites detected have read depths less than 10, a threshold used to identify SNP between strains of the highly monomorphic pathogen *Salmonella Paratyphi *A [36]. Furthermore, the analysis with a simulation procedure indicated that the read depth might contribute to the failure in this data set (Additional file 4**Table S4**). In the simulation, 1,000 Solexa data sets were created separately for the depth of 4, 6, 8 and 10. Each set consists of unique Solexa reads that were randomly sampled from the 38 Solexa reads covering SNP identified by Maq at the position of 395,176. All failed to pass the Maq filter (SNPfilter) except the data sets of the read depth of 10, where SNPs were detected in 99.9% of the simulated data. SNPfilter is one of the programs in the Maq package, which helps to filter SNPs according to predefined rules of the package.

Like many other computational tools, Maq analyses are local and all analyses narrow down to particular genome positions without considering global genome contexts. So, it is difficult to determine whether the heterogeneity is due to intra-genome variation or the heterogeneous population. This is exactly the case for five chromosomal positions detected in the gene-encoding putative transposase, where Maq identified them as heterozygotes (Additional file 5** Table S5**). The genotypic analysis indicated that they are indeed heterogeneous in the newly sequenced genome; however, they are also heterogeneous in the simulated sequence population from the isogenic reference genome, implying intra-genome variation at these sites (Additional file 6** Table S6**).

In the SNP analysis, Maq detects potential SNPs via comparing the consensus sequence to the reference genomes, which are then filtered by a set of predefined rules [35]. The filtering rules out SNPs if they are, among other constraints, falling in a possible repetitive region. It is understandable to rule out repetitive regions from SNP analysis due to possible intra-genome variations. The filtering, however, may have an unexpected consequence. As illustrated above, gene duplication and subsequent mutations are vital in pathogenic bacteria because they help the bacteria adapt to ever-changing environments [51]. By establishing and comparatively analyzing the heterogeneity genotypes at the newly sequenced strains as well as their isogenic reference strain, ***Gen^Ht^***can identify SNPs/heterogeneity sites that fall in the highly repetitive regions. This can be demonstrated in the comparative analysis between *S. aureus *JH9 and *S. aureus *JH1. Using the simulation procedure (See MATERIALS AND METHODS for detailed procedure), three differentiating loci were identified within the 23 S ribosomal RNA genes (Additional file 7** Fig. S1 **and Additional file 8** Table S7**). Among them, two loci are heterogeneous in JH1 but homogeneous in JH9.

### Future improvement

Despite the success of the demonstration, there are minor concerns in the development and application of ***Gen^Htr^***. First, ***Gen^Htr ^***utilized MegaBlast for the mapping of the Solexa reads and the identification of the genetic heterogeneity sites. The program uses a greedy algorithm for the nucleotide sequence-alignment search, up to 10 times faster than more common sequence-similarity programs [42]. The alignment tool is able to handle the massive data that Solexa generated but would sacrifice certain levels of accuracies. Besides its speed, another reason for choosing MegaBlast is its intolerance for mismatches between aligned sequences. For example, runs using MegaBlast with default MegaBlast parameters permit only two mismatches in an alignment of 37 base pairs, which allow the identification of Solexa reads that are likely to be orthologous to the reference genome. One of the alternative tools for aligning genome sequences with Solexa reads is Blat. A trial with the alignment tool indicated that it could provide much better alignments; for example, when the DNA fragment of the first 1,000 base pairs of the isogenic reference genome was run against the Solexa reads, MegaBlast achieved a full sequence alignment and a 100% sequence identity for 250 homologous Solexa reads while the number was much larger when Blat was applied -- 1,530 in total. As a result, genome coverage was increased. The average Solexa reads per position was up 12.3% from 130 to 146 when MegaBlast was replaced with Blat on the genome analysis of SRX07711. Blat was applied with default parameters, except with the option -out=blast. However, Blat took a much longer alignment time than MegaBlast, roughly 35 hours to align 2,873 ***R***eference ***G***enome ***D***NA ***F***ragments (***RGDFs) ***against the Solexa read data while MegaBlast took approximately 8 hours. On the other hand, Blat is much faster if Solexa reads were used as queries against the isogenic reference genome, taking only a few minutes to finish the analysis of the entire Solexa data. The problem is that substitutions could disrupt the alignments when they occur at sub-centric positions (Additional file 9** Table S8**). The sub-centric positions mean that the substitutions divide the read into a large DNA fragment of about 28 to 32 base pairs and a small fragment of 5-9 base pairs. Instead of making the alignment with the mismatches, Blat simply aligns the larger one, then searches downstream for the sequence identical to the smaller one and aligns with it. Approximately 81,135 such alignments were found when a Solexa data set was mapped to the USA300 isogenic reference genome. The result indicated that such analysis could compromise the SNP and heterogeneity analysis if not corrected.

Second, the sequencing errors are the main concerns. ***Gen^Htr ^***used the Phred value of 13 (base quality) as a measure to determine whether the base calls from Solexa reads are real or are derived from sequencing artifacts. However, a weighted function will be necessary. First, the quality scores are unevenly distributed along the 37-bp Solexa reads (Additional file 10** Table S9**). Overall, base calls at the 5' end of the Solexa read have a high-quality score that gradually decreases as sequencing reaches the 3' end [74]. For example, a majority of the base calls at the first position of these reads are high quality, where less than 5% of base calls have Phred values of less than 13. In the contrast, 10% of the base calls from position 2 to 13, 25% from 14 to 19, 50% from position 20 to 28, and 75% from position 29 to 37 are considered to be incorrect base calls based on the threshold. Therefore, the geographical location of the bases in the Solexa reads is an important factor in evaluating data quality. On the one hand, bases with a low Phred score are not necessarily wrong base calling. Indeed, previous experiments estimated that the error rate per base read of SNPs detected by Solexa and checked by Sanger sequencing for the Maq b5 (base quality ≥5) is 1.0 × 10^2^, indicating that the majority of the base calling with a low Phred score is correct. On the other hand, a higher base quality is not a guarantee of correct base calling. For the Maq b20 (base quality ≥20), the error rate is still 1.0 × 10^3 ^although tenfold lower. In addition, genotypes at some heterogeneity sites with a low Phred value showed the exact same patterns as those from the isogenic reference genome (Additional file 11** Table S10**). This also indicates that a base calling with a low-quality score can still be correct.

Another approach to evaluate the quality of the heterogeneity sites is to determine the general tendency of base substitutions. As described above, the progressive deterioration in quality scores as the sequencing proceeds leads to higher noise levels. The consequence is intensity more close to the background, which results in misleading base calling, e.g. it is more likely to be called T than A, or C than G [74]. If sequencing errors are the only cause of the heterogeneity, there should be more A- > T and G- > C substitutions than any other types of substitutions at the heterogeneity sites. Detailed examination of the heterogeneity genotypes found no such tendency. In Section III of table 1, there are more C- > A (23%) than any other substitutions. This is followed by G- > T (13.4%) and C- > G (11.5%). In fact, there are only 7% in both A- > T and G- > C substitutions, indicating that the resulting alternative nucleotides at the heterogeneity sites are not likely entirely from sequencing errors.

Third, the frequency of subpopulations in bacterial clone populations represents a great challenge in heterogeneity analysis. Rates from 10^-3 ^to 10^-6 ^of more highly vancomycin-resistant cells in hVISA cultures appear to define hVISA strains at the moment. It is certainly below the proven polymorphism detection limit. Evidence also indicated that the frequency, however, varied from strain to strain and condition to condition. Generally, strains with a higher level of drug-resistance appear to have higher frequencies of drug-resistant subpopulations when cultured at a medium with a lower drug concentration. For example, NYH-2*, an hVISA strain, has a minimum inhibitory concentration of 8 μg/ml. The frequency of drug-resistant subpopulations in NYH-2* is 100% at a vancomycin concentration of 2 μg/ml, 10% at 4 μg/ml and 6 × 10^-2 ^% at 8 μg/ml [6]. PC-3∗ another hVISA strain with a minimum inhibitory concentration of 16 μg/ml, has a drug-resistant subpopulation frequency of 100% when cultured at all three vancomycin concentrations: 2 μg/ml, 4 μg/ml, and 8 μg/ml [6]. Thus, a better experimental design plus greater genome coverage are expected to overcome the low polymorphism detection limit.

Finally, the complexity of bacterial genomes will make it difficult to assess the heterogeneity status of particular chromosomal positions if they occur in the DNA fragments with multiple copies, e.g. those observed in 5S/23 S ribosomal RNA and genes encoding sdrE protein and clumping factor A/B (Table 2). In these cases, the heterogeneity sites can be interpreted to be due either to mutations between paralogs (within the genome) or to mutation in orthologs (between genomes of different subpopulations). On the other hand, the heterogeneity sites detected in the single-copy DNA fragments are less problematic. These sites cover many positions in protein-encoding genes, including those at penicillin-binding protein 3, exonuclease RexB, DNA topoisomerase I, and DNA mismatch repair protein MutS, and involve in 48 different functions. It will be a rare event for all these genes to be duplicated in such a short period of time between the isogenic isolates. In addition, no recent duplication events are detected at this or the 14 other completely sequenced *S. aureus *genomes. This allows us to predict that the sites are truly heterogeneous, which likely represent multi-variants in the bacterial populations.

## Conclusions

In summary, ***Gen^Htr ^***was developed and tested with a newly sequenced *S. aureus *USA 300 cell line. Although it is much more time-consuming when compared to Maq, a popular tool for SNP analysis, ***Gen^Htr ^***is able to build genome-wide heterogeneity genotypes for both newly sequenced genomes (using massively parallel short-read sequencing) and their isogenic reference genome (using simulated data). From that, ***Gen^Htr ^***can predict potential multiple variants that pre-exist in the bacterial population as well as SNPs that occur in highly duplicated gene families. In addition, the establishment of genome-wide heterogeneity genotypes for newly sequenced genomes and their isogenic reference genomes allows the heterogeneity to be quantified. For example, we plan to use an evolving **d**istance (***d***) to quantify how many newly sequenced strains are evolved from the isogenic reference strains and characterize loci that have a greater complexity than those that happened in the 23 S rRNA genes. The successful implementation and testing of ***Gen^Htr ^***is expected to have a large impact on the research of bacterial pathogen. Rather than identifying sequence variations from strains to strains or isolates to isolates [75-81], the Solexa technology and ***Gen^Htr ^***will allow bacterial strains/isolates to be studied as heterogeneous populations instead of as monomorphic clones [5]. By this approach, the population dynamics of bacterial populations can be carefully characterized and comparatively analyzed with respect to genetic heterogeneity. With the paradigm shift, we expect that the evolutionary forces that shape bacterial populations can be evaluated at the DNA sequence level on the whole genome scale.

## Abbreviations

***Gen^Htr^***: Genetic heterogeneity analysis; ***RGDF***: Reference Genome DNA Fragment; ***Heterogeneity sites***: chromosomal positions with multiple variants of genes in the bacterial population; ***SNP***: Single-nucleotide polymorphism; ***IRG***: Isogenic reference genome; ***RFLP***: restriction fragment length polymorphisms; ***MIRU-VNTR***: spoligotyping and the mycobacterial interspersed repetitive-unit-variable-number tandem-repeat typing in the study of *M. tuberculosis *populations; ***MLST***: multilocus sequence typing; ***DLST***: double-locus sequence typing; ***PDIM***: Phthiocerol Dimycocerosate.

## Competing interests

The author declares that he has no competing interests.

## Authors' contributions

GX carried out software development, data analysis and manuscript preparation, and approved the final manuscript.

## Supplementary Material

Additional file 1 Table S1: Statistic of the new genome Click here for file

Additional file 2 Table S2: Copy number and heterogeneity in the simulated data from the referenced genome Click here for file

Additional file 3 Table S3: Distribution of "heterogeneity" sites at the isogenic reference genome. Click here for file

Additional file 4 Table S4: Affect of read depth on the performance of Maq in discovering SNPs. Click here for file

Additional file 5 Table S5: Heterogeneity and SNPs detected by Maq analysis. Click here for file

Additional file 6 **Table S6: Genotype analysis by *****Gen**^**Htr**^* Click here for file

Additional file 7 Table S7: Mutations detected in the *S. aureus *JH1 and JH9 by simulation Click here for file

Additional file 8 Fig. S1: Differences in mutations of 23 S ribosomal RNA between JH1 and JH9. Click here for file

Additional file 9 Table S8: Blat Alignment differences between two different models of analysis Click here for file

Additional file 10 Table S9: Percentile of Phred values on 37 positions on 5000 Solexa reads. Click here for file

Additional file 11 Table S10: A list of positions that are share same genotypes between the reference genome and those from the newly sequenced *S. aureus *subsp. USA300 cell line. Click here for file

## References

[B1] Feil EJ Maiden MC Achtman M Spratt BG The relative contributions of recombination and mutation to the divergence of clones of Neisseria meningitidis Mol Biol Evol 1999 16 1496 1502 10555280 10.1093/oxfordjournals.molbev.a026061

[B2] Feil EJ Smith JM Enright MC Spratt BG Estimating recombinational parameters in Streptococcus pneumoniae from multilocus sequence typing data Genetics 2000 154 1439 1450 10747043 10.1093/genetics/154.4.1439 PMC1461021

[B3] Lawrence JG Hendrickson H Lateral gene transfer: when will adolescence end? Mol Microbiol 2003 50 739 749 10.1046/j.1365-2958.2003.03778.x 14617137

[B4] Maynard Smith J Smith NH Detecting recombination from gene trees Mol Biol Evol 1998 15 590 599 9580989 10.1093/oxfordjournals.molbev.a025960

[B5] Jarzembowski T Wisniewska K Jozwik A Witkowski J Heterogeneity of methicillin-resistant Staphylococcus aureus strains (MRSA) characterized by flow cytometry Curr Microbiol 2009 59 78 80 10.1007/s00284-009-9395-x 19330377

[B6] Sieradzki K Roberts RB Haber SW Tomasz A The development of vancomycin resistance in a patient with methicillin-resistant Staphylococcus aureus infection N Engl J Med 1999 340 517 523 10.1056/NEJM199902183400704 10021472

[B7] Delgado A Riordan JT Lamichhane-Khadka R Winnett DC Jimenez J Robinson K O'Brien FG Cantore SA Gustafson JE Hetero-vancomycin-intermediate methicillin-resistant Staphylococcus aureus isolate from a medical center in Las Cruces, New Mexico J Clin Microbiol 2007 45 1325 1329 10.1128/JCM.02437-06 17267639 PMC1865829

[B8] O'Brien FG Lim TT Winnett DC Coombs GW Pearson JC Delgado A Langevin MJ Cantore SA Gonzalez L Gustafson JE Survey of methicillin-resistant Staphylococcus aureus strains from two hospitals in El Paso, Texas J Clin Microbiol 2005 43 2969 2972 10.1128/JCM.43.6.2969-2972.2005 15956434 PMC1151905

[B9] Post FA Willcox PA Mathema B Steyn LM Shean K Ramaswamy SV Graviss EA Shashkina E Kreiswirth BN Kaplan G Genetic polymorphism in Mycobacterium tuberculosis isolates from patients with chronic multidrug-resistant tuberculosis J Infect Dis 2004 190 99 106 10.1086/421501 15195248

[B10] Andreu N Gibert I Cell population heterogeneity in Mycobacterium tuberculosis H37Rv Tuberculosis (Edinb) 2008 88 553 559 10.1016/j.tube.2008.03.005 18502178

[B11] Matic I Radman M Taddei F Picard B Doit C Bingen E Denamur E Elion J Highly variable mutation rates in commensal and pathogenic Escherichia coli Science 1997 277 1833 1834 10.1126/science.277.5333.1833 9324769

[B12] LeClerc JE Payne WL Kupchella E Cebula TA Detection of mutator subpopulations in Salmonella typhimurium LT2 by reversion of his alleles Mutat Res 1998 400 89 97 9685594 10.1016/s0027-5107(98)00069-4

[B13] Richardson AR Yu Z Popovic T Stojiljkovic I Mutator clones of Neisseria meningitidis in epidemic serogroup A disease Proc Natl Acad Sci USA 2002 99 6103 6107 10.1073/pnas.092568699 11983903 PMC122909

[B14] Watson ME Jr Burns JL Smith AL Hypermutable Haemophilus influenzae with mutations in mutS are found in cystic fibrosis sputum Microbiology 2004 150 2947 2958 10.1099/mic.0.27230-0 15347753

[B15] Bjorkholm B Sjolund M Falk PG Berg OG Engstrand L Andersson DI Mutation frequency and biological cost of antibiotic resistance in Helicobacter pylori Proc Natl Acad Sci USA 2001 98 14607 14612 10.1073/pnas.241517298 11717398 PMC64729

[B16] del Campo R Morosini MI de la Pedrosa EG Fenoll A Munoz-Almagro C Maiz L Baquero F Canton R Population structure, antimicrobial resistance, mutation frequencies of Streptococcus pneumoniae isolates from cystic fibrosis patients J Clin Microbiol 2005 43 2207 2214 10.1128/JCM.43.5.2207-2214.2005 15872243 PMC1153755

[B17] Oliver A Levin BR Juan C Baquero F Blazquez J Hypermutation and the preexistence of antibiotic-resistant Pseudomonas aeruginosa mutants: implications for susceptibility testing and treatment of chronic infections Antimicrob Agents Chemother 2004 48 4226 4233 10.1128/AAC.48.11.4226-4233.2004 15504845 PMC525420

[B18] Enright MC Day NP Davies CE Peacock SJ Spratt BG Multilocus sequence typing for characterization of methicillin-resistant and methicillin-susceptible clones of Staphylococcus aureus J Clin Microbiol 2000 38 1008 1015 10698988 10.1128/jcm.38.3.1008-1015.2000 PMC86325

[B19] Melles DC Gorkink RF Boelens HA Snijders SV Peeters JK Moorhouse MJ van der Spek PJ van Leeuwen WB Simons G Verbrugh HA Natural population dynamics and expansion of pathogenic clones of Staphylococcus aureus J Clin Invest 2004 114 1732 1740 15599398 10.1172/JCI23083 PMC535072

[B20] Harmsen D Claus H Witte W Rothganger J Claus H Turnwald D Vogel U Typing of methicillin-resistant Staphylococcus aureus in a university hospital setting by using novel software for spa repeat determination and database management J Clin Microbiol 2003 41 5442 5448 10.1128/JCM.41.12.5442-5448.2003 14662923 PMC309029

[B21] Kuhn G Francioli P Blanc DS Evidence for clonal evolution among highly polymorphic genes in methicillin-resistant Staphylococcus aureus J Bacteriol 2006 188 169 178 10.1128/JB.188.1.169-178.2006 16352833 PMC1317586

[B22] Sakwinska O Kuhn G Balmelli C Francioli P Giddey M Perreten V Riesen A Zysset F Blanc DS Moreillon P Genetic diversity and ecological success of Staphylococcus aureus strains colonizing humans Appl Environ Microbiol 2009 75 175 183 10.1128/AEM.01860-08 18978084 PMC2612194

[B23] Silby MW Cerdeno-Tarraga AM Vernikos GS Giddens SR Jackson RW Preston GM Zhang XX Moon CD Gehrig SM Godfrey SA Genomic and genetic analyses of diversity and plant interactions of Pseudomonas fluorescens Genome Biol 2009 10 R51 10.1186/gb-2009-10-5-r51 19432983 PMC2718517

[B24] Shendure J Mitra RD Varma C Church GM Advanced sequencing technologies: methods and goals Nat Rev Genet 2004 5 335 344 10.1038/nrg1325 15143316

[B25] Srivatsan A Han Y Peng J Tehranchi AK Gibbs R Wang JD Chen R High-precision, whole-genome sequencing of laboratory strains facilitates genetic studies PLoS Genet 2008 4 e1000139 10.1371/journal.pgen.1000139 18670626 PMC2474695

[B26] Margulies M Egholm M Altman WE Attiya S Bader JS Bemben LA Berka J Braverman MS Chen YJ Chen Z Genome sequencing in microfabricated high-density picolitre reactors Nature 2005 437 376 380 16056220 10.1038/nature03959 PMC1464427

[B27] Tsibris AM Sagar M Gulick RM Su Z Hughes M Greaves W Subramanian M Flexner C Giguel F Leopold KE In vivo emergence of vicriviroc resistance in a human immunodeficiency virus type 1 subtype C-infected subject J Virol 2008 82 8210 8214 10.1128/JVI.00444-08 18495779 PMC2519584

[B28] Wang C Mitsuya Y Gharizadeh B Ronaghi M Shafer RW Characterization of mutation spectra with ultra-deep pyrosequencing: application to HIV-1 drug resistance Genome Res 2007 17 1195 1201 10.1101/gr.6468307 17600086 PMC1933516

[B29] Betts JC Dodson P Quan S Lewis AP Thomas PJ Duncan K McAdam RA Comparison of the proteome of Mycobacterium tuberculosis strain H37Rv with clinical isolate CDC 1551 Microbiology 2000 146 Pt 12 3205 3216 11101678 10.1099/00221287-146-12-3205

[B30] Brennan MJ Delogu G Chen Y Bardarov S Kriakov J Alavi M Jacobs WR Jr Evidence that mycobacterial PE_PGRS proteins are cell surface constituents that influence interactions with other cells Infect Immun 2001 69 7326 7333 10.1128/IAI.69.12.7326-7333.2001 11705904 PMC98818

[B31] Cole ST Brosch R Parkhill J Garnier T Churcher C Harris D Gordon SV Eiglmeier K Gas S Barry CE Deciphering the biology of Mycobacterium tuberculosis from the complete genome sequence Nature 1998 393 537 544 10.1038/31159 9634230

[B32] Poulet S Cole ST Characterization of the highly abundant polymorphic GC-rich-repetitive sequence (PGRS) present in Mycobacterium tuberculosis Arch Microbiol 1995 163 87 95 10.1007/BF00381781 7710330

[B33] Homer N BFAST: Blat-like Fast Accurate Search Tool 2009

[B34] Smith AD Xuan Z Zhang MQ Using quality scores and longer reads improves accuracy of Solexa read mapping BMC Bioinformatics 2008 9 128 10.1186/1471-2105-9-128 18307793 PMC2335322

[B35] Li H Ruan J Durbin R Mapping short DNA sequencing reads and calling variants using mapping quality scores Genome Res 2008 18 1851 1858 10.1101/gr.078212.108 18714091 PMC2577856

[B36] Holt KE Teo YY Li H Nair S Dougan G Wain J Parkhill J Detecting SNPs and estimating allele frequencies in clonal bacterial populations by sequencing pooled DNA Bioinformatics 2009 25 2074 2075 10.1093/bioinformatics/btp344 19497932 PMC2722999

[B37] Yu GX Snyder EE Boyle SM Crasta OR Czar M Mane SP Purkayastha A Sobral B Setubal JC A versatile computational pipeline for bacterial genome annotation improvement and comparative analysis, with Brucella as a use case Nucleic Acids Res 2007 35 3953 3962 10.1093/nar/gkm377 17553834 PMC1919506

[B38] Birney E Clamp M Durbin R GeneWise and Genomewise Genome Res 2004 14 988 995 10.1101/gr.1865504 15123596 PMC479130

[B39] de Boer AS Kremer K Borgdorff MW de Haas PE Heersma HF van Soolingen D Genetic heterogeneity in Mycobacterium tuberculosis isolates reflected in IS6110 restriction fragment length polymorphism patterns as low-intensity bands J Clin Microbiol 2000 38 4478 4484 11101583 10.1128/jcm.38.12.4478-4484.2000 PMC87624

[B40] Goldman RC Plumley KV Laughon BE The evolution of extensively drug resistant tuberculosis (XDR-TB): history, status and issues for global control Infect Disord Drug Targets 2007 7 73 91 10.2174/187152607781001844 17970220

[B41] Diep BA Gill SR Chang RF Phan TH Chen JH Davidson MG Lin F Lin J Carleton HA Mongodin EF Complete genome sequence of USA300, an epidemic clone of community-acquired meticillin-resistant Staphylococcus aureus Lancet 2006 367 731 739 10.1016/S0140-6736(06)68231-7 16517273

[B42] Zhang Z Schwartz S Wagner L Miller W A greedy algorithm for aligning DNA sequences J Comput Biol 2000 7 203 214 10.1089/10665270050081478 10890397

[B43] Kent WJ BLAT--the BLAST-like alignment tool Genome Res 2002 12 656 664 11932250 10.1101/gr.229202 PMC187518

[B44] Keightley PD Trivedi U Thomson M Oliver F Kumar S Blaxter ML Analysis of the genome sequences of three Drosophila melanogaster spontaneous mutation accumulation lines Genome Res 2009 19 1195 1201 10.1101/gr.091231.109 19439516 PMC2704435

[B45] Alouf JE Muller-Alouf H Staphylococcal and streptococcal superantigens: molecular, biological and clinical aspects Int J Med Microbiol 2003 292 429 440 10.1078/1438-4221-00232 12635926

[B46] Bhavsar AP Erdman LK Schertzer JW Brown ED Teichoic acid is an essential polymer in Bacillus subtilis that is functionally distinct from teichuronic acid J Bacteriol 2004 186 7865 7873 10.1128/JB.186.23.7865-7873.2004 15547257 PMC529093

[B47] Weidenmaier C Kokai-Kun JF Kristian SA Chanturiya T Kalbacher H Gross M Nicholson G Neumeister B Mond JJ Peschel A Role of teichoic acids in Staphylococcus aureus nasal colonization, a major risk factor in nosocomial infections Nat Med 2004 10 243 245 10.1038/nm991 14758355

[B48] Weidenmaier C Peschel A Xiong YQ Kristian SA Dietz K Yeaman MR Bayer AS Lack of wall teichoic acids in Staphylococcus aureus leads to reduced interactions with endothelial cells and to attenuated virulence in a rabbit model of endocarditis J Infect Dis 2005 191 1771 1777 10.1086/429692 15838806

[B49] Weidenmaier C Kokai-Kun JF Kulauzovic E Kohler T Thumm G Stoll H Gotz F Peschel A Differential roles of sortase-anchored surface proteins and wall teichoic acid in Staphylococcus aureus nasal colonization Int J Med Microbiol 2008 298 505 513 10.1016/j.ijmm.2007.11.006 18221914

[B50] Fraser JD Proft T The bacterial superantigen and superantigen-like proteins Immunol Rev 2008 225 226 243 10.1111/j.1600-065X.2008.00681.x 18837785

[B51] Tsuru T Kobayashi I Multiple genome comparison within a bacterial species reveals a unit of evolution spanning two adjacent genes in a tandem paralog cluster Mol Biol Evol 2008 25 2457 2473 10.1093/molbev/msn192 18765438 PMC2568036

[B52] Hakenbeck R Briese T Chalkley L Ellerbrok H Kalliokoski R Latorre C Leinonen M Martin C Antigenic variation of penicillin-binding proteins from penicillin-resistant clinical strains of Streptococcus pneumoniae J Infect Dis 1991 164 313 319 1856480 10.1093/infdis/164.2.313

[B53] Hakenbeck R Briese T Chalkley L Ellerbrok H Kalliokoski R Latorre C Leinonen M Martin C Variability of penicillin-binding proteins from penicillin-sensitive Streptococcus pneumoniae J Infect Dis 1991 164 307 312 1856479 10.1093/infdis/164.2.307

[B54] Martin C Briese T Hakenbeck R Nucleotide sequences of genes encoding penicillin-binding proteins from Streptococcus pneumoniae and Streptococcus oralis with high homology to Escherichia coli penicillin-binding proteins 1a and 1b J Bacteriol 1992 174 4517 4523 1624444 10.1128/jb.174.13.4517-4523.1992 PMC206242

[B55] Fitzgerald JR Loughman A Keane F Brennan M Knobel M Higgins J Visai L Speziale P Cox D Foster TJ Fibronectin-binding proteins of Staphylococcus aureus mediate activation of human platelets via fibrinogen and fibronectin bridges to integrin GPIIb/IIIa and IgG binding to the FcgammaRIIa receptor Mol Microbiol 2006 59 212 230 10.1111/j.1365-2958.2005.04922.x 16359330

[B56] Komatsuzawa H Ohta K Fujiwara T Choi GH Labischinski H Sugai M Cloning and sequencing of the gene, fmtC, which affects oxacillin resistance in methicillin-resistant Staphylococcus aureus FEMS Microbiol Lett 2001 203 49 54 10.1111/j.1574-6968.2001.tb10819.x 11557139

[B57] Muthaiyan A Silverman JA Jayaswal RK Wilkinson BJ Transcriptional profiling reveals that daptomycin induces the Staphylococcus aureus cell wall stress stimulon and genes responsive to membrane depolarization Antimicrob Agents Chemother 2008 52 980 990 10.1128/AAC.01121-07 18086846 PMC2258546

[B58] Kovacikova G Skorupski K A Vibrio cholerae LysR homolog, AphB, cooperates with AphA at the tcpPH promoter to activate expression of the ToxR virulence cascade J Bacteriol 1999 181 4250 4256 10400582 10.1128/jb.181.14.4250-4256.1999 PMC93926

[B59] Sperandio B Gautier C McGovern S Ehrlich DS Renault P Martin-Verstraete I Guedon E Control of methionine synthesis and uptake by MetR and homocysteine in Streptococcus mutans J Bacteriol 2007 189 7032 7044 10.1128/JB.00703-07 17675375 PMC2045202

[B60] Springer B Sander P Sedlacek L Hardt WD Mizrahi V Schar P Bottger EC Lack of mismatch correction facilitates genome evolution in mycobacteria Mol Microbiol 2004 53 1601 1609 10.1111/j.1365-2958.2004.04231.x 15341642

[B61] Smania AM Segura I Pezza RJ Becerra C Albesa I Argarana CE Emergence of phenotypic variants upon mismatch repair disruption in Pseudomonas aeruginosa Microbiology 2004 150 1327 1338 10.1099/mic.0.26751-0 15133095

[B62] Prunier AL Leclercq R Role of mutS and mutL genes in hypermutability and recombination in Staphylococcus aureus J Bacteriol 2005 187 3455 3464 10.1128/JB.187.10.3455-3464.2005 15866932 PMC1112015

[B63] Sniegowski PD Gerrish PJ Lenski RE Evolution of high mutation rates in experimental populations of E. coli Nature 1997 387 703 705 10.1038/42701 9192894

[B64] LeClerc JE Li B Payne WL Cebula TA High mutation frequencies among Escherichia coli and Salmonella pathogens Science 1996 274 1208 1211 10.1126/science.274.5290.1208 8895473

[B65] Oliver A Baquero F Blazquez J The mismatch repair system (mutS, mutL and uvrD genes) in Pseudomonas aeruginosa: molecular characterization of naturally occurring mutants Mol Microbiol 2002 43 1641 1650 10.1046/j.1365-2958.2002.02855.x 11952911

[B66] Rocha EP Cornet E Michel B Comparative and evolutionary analysis of the bacterial homologous recombination systems PLoS Genet 2005 1 e15 10.1371/journal.pgen.0010015 16132081 PMC1193525

[B67] Dorman CJ Corcoran CP Bacterial DNA topology and infectious disease Nucleic Acids Res 2009 37 672 678 10.1093/nar/gkn996 19073701 PMC2647292

[B68] Canu A Malbruny B Coquemont M Davies TA Appelbaum PC Leclercq R Diversity of ribosomal mutations conferring resistance to macrolides, clindamycin, streptogramin, telithromycin in Streptococcus pneumoniae Antimicrob Agents Chemother 2002 46 125 131 10.1128/AAC.46.1.125-131.2002 11751122 PMC126998

[B69] Prunier AL Malbruny B Tande D Picard B Leclercq R Clinical isolates of Staphylococcus aureus with ribosomal mutations conferring resistance to macrolides Antimicrob Agents Chemother 2002 46 3054 3056 10.1128/AAC.46.9.3054-3056.2002 12183270 PMC127407

[B70] Palmqvist N Josefsson E Tarkowski A Clumping factor A-mediated virulence during Staphylococcus aureus infection is retained despite fibrinogen depletion Microbes Infect 2004 6 196 201 10.1016/j.micinf.2003.10.014 14998518

[B71] Ni Eidhin D Perkins S Francois P Vaudaux P Hook M Foster TJ Clumping factor B (ClfB), a new surface-located fibrinogen-binding adhesin of Staphylococcus aureus Mol Microbiol 1998 30 245 257 10.1046/j.1365-2958.1998.01050.x 9791170

[B72] Schaffer AC Solinga RM Cocchiaro J Portoles M Kiser KB Risley A Randall SM Valtulina V Speziale P Walsh E Immunization with Staphylococcus aureus clumping factor B, a major determinant in nasal carriage, reduces nasal colonization in a murine model Infect Immun 2006 74 2145 2153 10.1128/IAI.74.4.2145-2153.2006 16552044 PMC1418917

[B73] Corrigan RM Miajlovic H Foster TJ Surface proteins that promote adherence of Staphylococcus aureus to human desquamated nasal epithelial cells BMC Microbiol 2009 9 22 10.1186/1471-2180-9-22 19183486 PMC2642834

[B74] Rougemont J Amzallag A Iseli C Farinelli L Xenarios I Naef F Probabilistic base calling of Solexa sequencing data BMC Bioinformatics 2008 9 431 10.1186/1471-2105-9-431 18851737 PMC2575221

[B75] Sreevatsan S Pan X Stockbauer KE Connell ND Kreiswirth BN Whittam TS Musser JM Restricted structural gene polymorphism in the Mycobacterium tuberculosis complex indicates evolutionarily recent global dissemination Proc Natl Acad Sci USA 1997 94 9869 9874 10.1073/pnas.94.18.9869 9275218 PMC23284

[B76] Didelot X Falush D Inference of Bacterial Microevolution Using Multilocus Sequence Data Genetics 2007 175 1251 1266 10.1534/genetics.106.063305 17151252 PMC1840087

[B77] Fitzgerald JR Sturdevant DE Mackie SM Gill SR Musser JM Evolutionary genomics of Staphylococcus aureus: insights into the origin of methicillin-resistant strains and the toxic shock syndrome epidemic Proc Natl Acad Sci USA 2001 98 8821 8826 10.1073/pnas.161098098 11447287 PMC37519

[B78] Maiden MC Bygraves JA Feil E Morelli G Russell JE Urwin R Zhang Q Zhou J Zurth K Caugant DA Multilocus sequence typing: a portable approach to the identification of clones within populations of pathogenic microorganisms Proc Natl Acad Sci USA 1998 95 3140 3145 10.1073/pnas.95.6.3140 9501229 PMC19708

[B79] Salama N Guillemin K McDaniel TK Sherlock G Tompkins L Falkow S A whole-genome microarray reveals genetic diversity among Helicobacter pylori strains Proc Natl Acad Sci USA 2000 97 14668 14673 10.1073/pnas.97.26.14668 11121067 PMC18976

[B80] Urwin R Maiden MC Multi-locus sequence typing: a tool for global epidemiology Trends Microbiol 2003 11 479 487 10.1016/j.tim.2003.08.006 14557031

[B81] Maiden MC Bygraves JA Feil E Morelli G Russell JE Urwin R Zhang Q Zhou J Zurth K Caugant DA Multilocus sequence typing: a portable approach to the identification of clones within populations of pathogenic microorganisms Proc Natl Acad Sci USA 1998 95 3140 3145 10.1073/pnas.95.6.3140 9501229 PMC19708

