# Supplementary material for: GenHtr: a tool for comparative assessment of genetic heterogeneity in microbial genomes generated by massive short-read sequencing
Source: BMC Bioinformatics. 2010 Oct 12;11:508. doi: 10.1186/1471-2105-11-508 (PMC2967562; doi:10.1186/1471-2105-11-508)
Supplement: Additional file 1 — Table S1: Statistic of the new genome [file 1471-2105-11-508-S1.DOC]

**Additional file 1 Table S1**. Statistic of the new genome

| The Number of gaps: **409**  Gap Percentage: **0.014%**  Filled Positions: **2872360**  Average reads sampled per position: **130.09**  Total number of positions at the referenced genome:**2872769**  Maximum number of reads sampled per position:**1346** | | | |
| --- | --- | --- | --- |
| **Number of reads Sequenced**  **Per Chromosomal Position** | **Number of Positions** | **Percentage** | **Accumulated Percentage**  **(From highest to Lowest)** |
| >1200 | 310 | 0.01079 | 0.01079 |
| >=500 & <1200 | 18794 | 0.65435 | 0.66515 |
| >=200 & <500 | 78186 | 2.72223 | 3.38738 |
| >=100 & <200 | 2125085 | 73.9899 | 77.3773 |
| >=50 & <100 | 604098 | 21.0331 | 98.4105 |
| >=20 & <50 | 42074 | 1.46490 | 99.8754 |
| >=10 & <20 | 3193 | 0.11117 | 99.9865 |
| <10 & >=5 | 385 | 0.01340 | 100 |
