# Supplementary material for: GenHtr: a tool for comparative assessment of genetic heterogeneity in microbial genomes generated by massive short-read sequencing
Source: BMC Bioinformatics. 2010 Oct 12;11:508. doi: 10.1186/1471-2105-11-508 (PMC2967562; doi:10.1186/1471-2105-11-508)
Supplement: Additional file 2 — Table S2: Copy number and heterogeneity in the simulated data from the referenced genome [file 1471-2105-11-508-S2.DOC]

**Additional file 2** **Table S2.** Copy number and heterogeneity in the simulated data from the referenced genome

| **Chrom**  **Position** | **Gene and Function** | **Genotype**  **at**  **NC_007793** | **Alignment with the genome of S. aureus subsp. USA300 FPR3757** | **Copy Number** |
| --- | --- | --- | --- | --- |
| 2690087 | antibiotic transport-associated protein-like protein | T:37 | **>gi|87159884|ref|NC_007793.1| Staphylococcus aureus subsp. aureus  USA300_FPR3757, complete genome  Length = 2872769   Score = 65.9 bits (33), Expect = 1e-12  Identities = 36/37 (97%)  Strand = Plus / Plus    Query: 1 tgcttgttgttttgaatttagttgatgcttgttttga 37  |||||||| |||||||||||||||||||||||||||| Sbjct: 2690079 tgcttgtttttttgaatttagttgatgcttgttttga 2690115    Database: NC_007790.1.2.3_DB.fna  Posted date: Jan 3, 2010 9:09 PM  Number of letters in database: 2,917,469  Number of sequences in database: 4 2690087** | Single copy |
| 408863 | 5-methyltetrahydropteroyltriglutamate--homocysteine S-methyltransferase | G:37 | **>gi|87159884|ref|NC_007793.1| Staphylococcus aureus subsp. aureus  USA300_FPR3757, complete genome  Length = 2872769   Score = 65.9 bits (33), Expect = 1e-12  Identities = 36/37 (97%)  Strand = Plus / Plus    Query: 1 gtaatttttctccgaagaattcaaccatttcattacg 37  |||||||||||||||||||||||||||| |||||||| Sbjct: 408835 gtaatttttctccgaagaattcaaccatgtcattacg 408871    Database: NC_007790.1.2.3_DB.fna  Posted date: Jan 3, 2010 9:09 PM  Number of letters in database: 2,917,469  Number of sequences in database: 4 408863** | Single copy |
| 1180638 | cell division protein ftsA | G:37 | **>gi|87159884|ref|NC_007793.1| Staphylococcus aureus subsp. aureus**  **USA300_FPR3757, complete genome**  **Length = 2872769**  **Score = 65.9 bits (33), Expect = 1e-12**  **Identities = 36/37 (97%)**  **Strand = Plus / Plus**    **Query: 1 gtgttgatttattagatgtttactctgatgcatataa 37**  **|||||||| ||||||||||||||||||||||||||||**  **Sbjct: 1180630 gtgttgatgtattagatgtttactctgatgcatataa 1180666**  **Database: NC_007790.1.2.3_DB.fna**  **Posted date: Jan 3, 2010 9:09 PM**  **Number of letters in database: 2,917,469**  **Number of sequences in database: 4** | Single copy |
| 1714319 | acetyl-CoA carboxylase, biotin carboxyl carrier protein | C:37 | **>gi|87159884|ref|NC_007793.1| Staphylococcus aureus subsp. aureus  USA300_FPR3757, complete genome  Length = 2872769   Score = 65.9 bits (33), Expect = 1e-12  Identities = 36/37 (97%)  Strand = Plus / Plus    Query: 1 cttttatcgcttcaacatatcctataatatctccctt 37  |||| |||||||||||||||||||||||||||||||| Sbjct: 1714315 ctttcatcgcttcaacatatcctataatatctccctt 1714351    Database: NC_007790.1.2.3_DB.fna  Posted date: Jan 3, 2010 9:09 PM  Number of letters in database: 2,917,469  Number of sequences in database: 4** | Single copy |
| 2638027 | gluconate kinase | C:36 | **>gi|87159884|ref|NC_007793.1| Staphylococcus aureus subsp. aureus  USA300_FPR3757, complete genome  Length = 2872769   Score = 65.9 bits (33), Expect = 1e-12  Identities = 36/37 (97%)  Strand = Plus / Plus    Query: 1 atctgaaatcatttgacgccatacttcacttttcgca 37  |||||| |||||||||||||||||||||||||||||| Sbjct: 2638021 atctgacatcatttgacgccatacttcacttttcgca 2638057    Database: NC_007790.1.2.3_DB.fna  Posted date: Jan 3, 2010 9:09 PM  Number of letters in database: 2,917,469  Number of sequences in database: 4 2638027** | Single copy |
| 2481059 | response regulator protein | G:37 | **>gi|87159884|ref|NC_007793.1| Staphylococcus aureus subsp. aureus**  **USA300_FPR3757, complete genome**  **Length = 2872769**  **Score = 65.9 bits (33), Expect = 1e-12**  **Identities = 36/37 (97%)**  **Strand = Plus / Plus**    **Query: 1 cgatattgcagtggtagatattatgatggatgttatg 37**  **|||||||||||||||||||||||||||||||| ||||**  **Sbjct: 2481027 cgatattgcagtggtagatattatgatggatggtatg 2481063**  **Database: NC_007790.1.2.3_DB.fna**  **Posted date: Jan 3, 2010 9:09 PM**  **Number of letters in database: 2,917,469**  **Number of sequences in database: 4** | Single copy |
| 2212436 | thiamine-phosphate pyrophosphorylase | C:37 | **>gi|87159884|ref|NC_007793.1| Staphylococcus aureus subsp. aureus  USA300_FPR3757, complete genome  Length = 2872769   Score = 65.9 bits (33), Expect = 1e-12  Identities = 36/37 (97%)  Strand = Plus / Minus    Query: 1 aaaattgaaaatattgaaaagactgttaatcgattca 37  |||| |||||||||||||||||||||||||||||||| Sbjct: 2212440 aaaagtgaaaatattgaaaagactgttaatcgattca 2212404    Database: NC_007790.1.2.3_DB.fna  Posted date: Jan 3, 2010 9:09 PM  Number of letters in database: 2,917,469  Number of sequences in database: 4 2212436** | Single copy |
| 1252956 | DNA topoisomerase I | G:37 | **>gi|87159884|ref|NC_007793.1| Staphylococcus aureus subsp. aureus  USA300_FPR3757, complete genome  Length = 2872769   Score = 65.9 bits (33), Expect = 1e-12  Identities = 36/37 (97%)  Strand = Plus / Plus    Query: 1 gtccttttgtaaaagaattgaaaaaacatgcaaaaaa 37  ||||| ||||||||||||||||||||||||||||||| Sbjct: 1252951 gtcctgttgtaaaagaattgaaaaaacatgcaaaaaa 1252987    Database: NC_007790.1.2.3_DB.fna  Posted date: Jan 3, 2010 9:09 PM  Number of letters in database: 2,917,469  Number of sequences in database: 4 1252956** | Single copy |
| 1948255 | lantibiotic epidermin leader peptide processing serine protease EpiP | C:37 | **>gi|87159884|ref|NC_007793.1| Staphylococcus aureus subsp. aureus**  **USA300_FPR3757, complete genome**  **Length = 2872769**  **Score = 65.9 bits (33), Expect = 1e-12**  **Identities = 36/37 (97%)**  **Strand = Plus / Minus**    **Query: 1 atctatgtacaatgtcgttacagtaggatctacagat 37**  **||||||| |||||||||||||||||||||||||||||**  **Sbjct: 1948262 atctatggacaatgtcgttacagtaggatctacagat 1948226**  **Database: NC_007790.1.2.3_DB.fna**  **Posted date: Jan 3, 2010 9:09 PM**  **Number of letters in database: 2,917,469**  **Number of sequences in database: 4** | Single copy |
| 1309034 | DNA mismatch repair protein MutS | C:37 | **>gi|87159884|ref|NC_007793.1| Staphylococcus aureus subsp. aureus  USA300_FPR3757, complete genome  Length = 2872769   Score = 65.9 bits (33), Expect = 1e-12  Identities = 36/37 (97%)  Strand = Plus / Minus    Query: 1 gtaaaaaatcatcgatatcaaatttaaaaatggtcg 36**  **||||||||||||||||||||||||||||||||||||**  **Sbjct: 1309016 gtaaaaaatcatcgatatcaaatttaaaaatggtcg 1309051    Database: NC_007790.1.2.3_DB.fna  Posted date: Jan 3, 2010 9:09 PM  Number of letters in database: 2,917,469  Number of sequences in database: 4** | Single copy |
| 950365 | exonuclease RexB | C:37 | **>gi|87159884|ref|NC_007793.1| Staphylococcus aureus subsp. aureus  USA300_FPR3757, complete genome  Length = 2872769   Score = 61.9 bits (31), Expect = 1e-11  Identities = 34/35 (97%)  Strand = Plus / Plus    Query: 1 acagaaggctaatgtcgagttaagtccaacatcag 35  |||| |||||||||||||||||||||||||||||| Sbjct: 950361 acagcaggctaatgtcgagttaagtccaacatcag 950395    Database: NC_007790.1.2.3_DB.fna  Posted date: Jan 3, 2010 9:09 PM  Number of letters in database: 2,917,469  Number of sequences in database: 4 950365** | Single copy |
| 861340 | clumping factor A | C:49 | **>gi|87159884|ref|NC_007793.1| Staphylococcus aureus subsp. aureus  USA300_FPR3757, complete genome  Length = 2872769   Score = 65.9 bits (33), Expect = 1e-12  Identities = 36/37 (97%)  Strand = Plus / Plus   Query: 1 gattcagattcagatagcgattcagattctgacagtg 37  ||||||||||||||||||||||||||||| ||||||| Sbjct: 861131 gattcagattcagatagcgattcagattccgacagtg 861167   Score = 65.9 bits (33), Expect = 1e-12  Identities = 36/37 (97%)  Strand = Plus / Plus   Query: 1 gattcagattcagatagcgattcagattctgacagtg 37  ||||||||||||||||||||||||||||| ||||||| Sbjct: 861311 gattcagattcagatagcgattcagattccgacagtg 861347   Score = 61.9 bits (31), Expect = 2e-11  Identities = 34/35 (97%)  Strand = Plus / Plus   Query: 1 gattcagattcagatagcgattcagattctgacag 35  ||||||||||||||||||||||||||||| ||||| Sbjct: 617825 gattcagattcagatagcgattcagattcagacag 617859    Database: NC_007790.1.2.3_DB.fna  Posted date: Jan 3, 2010 9:09 PM  Number of letters in database: 2,917,469  Number of sequences in database: 4 861340** | 3 identical copies |
| 561501 | 5S ribosomal RNA | T:229 | **>gi|87159884|ref|NC_007793.1| Staphylococcus aureus subsp. aureus  USA300_FPR3757, complete genome  Length = 2872769   Score = 65.9 bits (33), Expect = 1e-12  Identities = 36/37 (97%)  Strand = Plus / Plus   Query: 1 cgtcgatggtagtcgaacttacgttccgccagagtag 37  ||||||||||||||||||||||||||||| ||||||| Sbjct: 517869 cgtcgatggtagtcgaacttacgttccgctagagtag 517905**  **Score = 65.9 bits (33), Expect = 1e-12  Identities = 36/37 (97%)  Strand = Plus / Plus   Query: 1 cgtcgatggtagtcgaacttacgttccgccagagtag 37  ||||||||||||||||||||||||||||| ||||||| Sbjct: 556262 cgtcgatggtagtcgaacttacgttccgctagagtag 556298   Score = 65.9 bits (33), Expect = 1e-12  Identities = 36/37 (97%)  Strand = Plus / Plus   Query: 1 cgtcgatggtagtcgaacttacgttccgccagagtag 37  ||||||||||||||||||||||||||||| ||||||| Sbjct: 561472 cgtcgatggtagtcgaacttacgttccgctagagtag 561508   Score = 65.9 bits (33), Expect = 1e-12  Identities = 36/37 (97%)  Strand = Plus / Minus    Query: 1 cgtcgatggtagtcgaacttacgttccgccagagtag 37  ||||||||||||||||||||||||||||| ||||||| Sbjct: 1997636 cgtcgatggtagtcgaacttacgttccgctagagtag 1997600**  **Query: 1 cgtcgatggtagtcgaacttacgttccgccagagtag 37  ||||||||||||||||||||||||||||| ||||||| Sbjct: 2175860 cgtcgatggtagtcgaacttacgttccgctagagtag 2175824   Score = 65.9 bits (33), Expect = 1e-12  Identities = 36/37 (97%)  Strand = Plus / Minus   Query: 1 cgtcgatggtagtcgaacttacgttccgccagagtag 37  ||||||||||||||||||||||||||||| ||||||| Sbjct: 2292414 cgtcgatggtagtcgaacttacgttccgctagagtag 2292378    Database: NC_007790.1.2.3_DB.fna  Posted date: Jan 3, 2010 9:09 PM  Number of letters in database: 2,917,469  Number of sequences in database: 4** | 6 identical copies |
| 613762 | sdrC protein | A:5 G:97 | **>gi|87159884|ref|NC_007793.1| Staphylococcus aureus subsp. aureus**  **USA300_FPR3757, complete genome**  **Length = 2872769**  **Score = 73.8 bits (37), Expect = 4e-15**  **Identities = 37/37 (100%)**  **Strand = Plus / Minus**    **Query: 1 acagcgactcagattcagatagcgattcagactcaga 37**  **|||||||||||||||||||||||||||||||||||||**  **Sbjct: 2774920 acagcgactcagattcagatagcgattcagactcaga 2774884**  **Score = 69.9 bits (35), Expect = 6e-14**  **Identities = 35/35 (100%)**  **Strand = Plus / Plus**    **Query: 3 agcgactcagattcagatagcgattcagactcaga 37**  **|||||||||||||||||||||||||||||||||||**  **Sbjct: 621860 agcgactcagattcagatagcgattcagactcaga 621894**  **Score = 69.9 bits (35), Expect = 6e-14**  **Identities = 35/35 (100%)**  **Strand = Plus / Minus**    **Query: 3 agcgactcagattcagatagcgattcagactcaga 37**  **|||||||||||||||||||||||||||||||||||**  **Sbjct: 2774846 agcgactcagattcagatagcgattcagactcaga 2774812**  **Score = 65.9 bits (33), Expect = 1e-12**  **Identities = 36/37 (97%)**  **Strand = Plus / Plus**  **Query: 1 acagcgactcagattcagatagcgattcagactcaga 37**  **|||||||||||||||||||||||||||| ||||||||**  **Sbjct: 613734 acagcgactcagattcagatagcgattcggactcaga 613770**  **Score = 65.9 bits (33), Expect = 1e-12**  **Identities = 36/37 (97%)**  **Strand = Plus / Plus**    **Query: 1 acagcgactcagattcagatagcgattcagactcaga 37**  **|||||||||||||||||||||||||||| ||||||||**  **Sbjct: 621894 acagcgactcagattcagatagcgattcggactcaga 621930**  **Score = 65.9 bits (33), Expect = 1e-12**  **Identities = 36/37 (97%)**  **Strand = Plus / Plus**    **Query: 1 acagcgactcagattcagatagcgattcagactcaga 37**  **|||||||||||||||||||||||||||| ||||||||**  **Sbjct: 621966 acagcgactcagattcagatagcgattcggactcaga 622002**  **Score = 65.9 bits (33), Expect = 1e-12**  **Identities = 36/37 (97%)**  **Strand = Plus / Minus**    **Query: 1 acagcgactcagattcagatagcgattcagactcaga 37**  **|||||||||||||||||||||||||||| ||||||||**  **Sbjct: 2774812 acagcgactcagattcagatagcgattcggactcaga 2774776**  **Score = 61.9 bits (31), Expect = 2e-11**  **Identities = 34/35 (97%)**  **Strand = Plus / Minus**  **Query: 3 agcgactcagattcagatagcgattcagactcaga 37**  **||||||||||||||||||||||||||||| |||||**  **Sbjct: 2775188 agcgactcagattcagatagcgattcagattcaga 2775154**  **Database: NC_007790.1.2.3_DB.fna**  **Posted date: Jan 3, 2010 9:09 PM**  **Number of letters in database: 2,917,469**  **Number of sequences in database: 4** | 8 copies: the 3 with “A” and 5 with “G” |
| 621505 | sdrE protein | A:67 T:5 | **>gi|87159884|ref|NC_007793.1| Staphylococcus aureus subsp. aureus  USA300_FPR3757, complete genome  Length = 2872769   Score = 71.9 bits (36), Expect = 2e-14  Identities = 36/36 (100%)  Strand = Plus / Plus    Query: 1 ttgcaaaacgaaaaaggcgaagtaattggtacaact 36  |||||||||||||||||||||||||||||||||||| Sbjct: 613058 ttgcaaaacgaaaaaggcgaagtaattggtacaact 613093     Score = 63.9 bits (32), Expect = 4e-12  Identities = 35/36 (97%)  Strand = Plus / Plus    Query: 1 ttgcaaaacgaaaaaggcgaagtaattggtacaact 36  ||||||||||||||||||||||||||||| |||||| Sbjct: 621476 ttgcaaaacgaaaaaggcgaagtaattggaacaact 621511    Database: NC_007790.1.2.3_DB.fna  Posted date: Jan 3, 2010 9:09 PM  Number of letters in database: 2,917,469  Number of sequences in database: 4** | 2 copies: 1 with “A” and 1 with “T”. |
| 2633516 | fibronectin binding protein A | A:37 C:5 | **>gi|87159884|ref|NC_007793.1| Staphylococcus aureus subsp. aureus**  **USA300_FPR3757, complete genome**  **Length = 2872769**  **Score = 73.8 bits (37), Expect = 4e-15**  **Identities = 37/37 (100%)**  **Strand = Plus / Minus**    **Query: 1 aaccgaaatatgaacaaggtggcaatatcgtagatat 37**  **|||||||||||||||||||||||||||||||||||||**  **Sbjct: 2629974 aaccgaaatatgaacaaggtggcaatatcgtagatat 2629938**  **Score = 65.9 bits (33), Expect = 1e-12**  **Identities = 36/37 (97%)**  **Strand = Plus / Minus**    **Query: 1 aaccgaaatatgaacaaggtggcaatatcgtagatat 37**  **|||| ||||||||||||||||||||||||||||||||**  **Sbjct: 2633520 aacctaaatatgaacaaggtggcaatatcgtagatat 2633484**  **Database: NC_007790.1.2.3_DB.fna**  **Posted date: Jan 3, 2010 9:09 PM**  **Number of letters in database: 2,917,469**  **Number of sequences in database: 4** | 2 copies: 1 with “A” and 1 with “C”. |
| 613639 | sdrC protein | T:164 C:4 | **>gi|87159884|ref|NC_007793.1| Staphylococcus aureus subsp. aureus  USA300_FPR3757, complete genome  Length = 2872769   Score = 73.8 bits (37), Expect = 4e-15  Identities = 37/37 (100%)  Strand = Plus / Plus    Query: 1 actcagactcagacagcgactcagattcagatagcga 37  ||||||||||||||||||||||||||||||||||||| Sbjct: 621954 actcagactcagacagcgactcagattcagatagcga 621990     Score = 73.8 bits (37), Expect = 4e-15  Identities = 37/37 (100%)  Strand = Plus / Minus    Query: 1 actcagactcagacagcgactcagattcagatagcga 37  ||||||||||||||||||||||||||||||||||||| Sbjct: 2774932 actcagactcagacagcgactcagattcagatagcga 2774896     Score = 69.9 bits (35), Expect = 6e-14  Identities = 35/35 (100%)  Strand = Plus / Plus    Query: 3 tcagactcagacagcgactcagattcagatagcga 37  ||||||||||||||||||||||||||||||||||| Sbjct: 621884 tcagactcagacagcgactcagattcagatagcga 621918     Score = 69.9 bits (35), Expect = 6e-14  Identities = 35/35 (100%)  Strand = Plus / Minus    Query: 3 tcagactcagacagcgactcagattcagatagcga 37  ||||||||||||||||||||||||||||||||||| Sbjct: 2774822 tcagactcagacagcgactcagattcagatagcga 2774788     Score = 61.9 bits (31), Expect = 2e-11  Identities = 34/35 (97%)  Strand = Plus / Plus Query: 3 tcagactcagacagcgactcagattcagatagcga 37  ||||| ||||||||||||||||||||||||||||| Sbjct: 613634 tcagattcagacagcgactcagattcagatagcga 613668     Score = 61.9 bits (31), Expect = 2e-11  Identities = 34/35 (97%)  Strand = Plus / Plus    Query: 3 tcagactcagacagcgactcagattcagatagcga 37  ||||| ||||||||||||||||||||||||||||| Sbjct: 613724 tcagattcagacagcgactcagattcagatagcga 613758     Score = 61.9 bits (31), Expect = 2e-11  Identities = 34/35 (97%)  Strand = Plus / Plus    Query: 3 tcagactcagacagcgactcagattcagatagcga 37  ||||| ||||||||||||||||||||||||||||| Sbjct: 621794 tcagattcagacagcgactcagattcagatagcga 621828    Database: NC_007790.1.2.3_DB.fna  Posted date: Jan 3, 2010 9:09 PM  Number of letters in database: 2,917,469  Number of sequences in database: 4** | 7 copies: 4 with “T” and 3 with “C”. |
| 618130 | sdrD protein | A:89 C:5 G:2 | **>gi|87159884|ref|NC_007793.1| Staphylococcus aureus subsp. aureus**  **USA300_FPR3757, complete genome**  **Length = 2872769**  **Score = 73.8 bits (37), Expect = 4e-15**  **Identities = 37/37 (100%)**  **Strand = Plus / Minus**    **Query: 1 tgtctgaatctgagtcgctatctgagtcggaatcgct 37**  **|||||||||||||||||||||||||||||||||||||**  **Sbjct: 861290 tgtctgaatctgagtcgctatctgagtcggaatcgct 861254**  **Score = 65.9 bits (33), Expect = 1e-12**  **Identities = 36/37 (97%)**  **Strand = Plus / Minus**    **Query: 1 tgtctgaatctgagtcgctatctgagtcggaatcgct 37**  **|||||||||||||||||||||||||||| ||||||||**  **Sbjct: 622022 tgtctgaatctgagtcgctatctgagtccgaatcgct 621986**  **Score = 60.0 bits (30), Expect = 6e-11**  **Identities = 33/34 (97%)**  **Strand = Plus / Minus**    **Query: 1 tgtctgaatctgagtcgctatctgagtcggaatc 34**  **|||||||||||||||||||||||||||| |||||**  **Sbjct: 617906 tgtctgaatctgagtcgctatctgagtctgaatc 617873**  **Score = 60.0 bits (30), Expect = 6e-11**  **Identities = 33/34 (97%)**  **Strand = Plus / Minus**    **Query: 1 tgtctgaatctgagtcgctatctgagtcggaatc 34**  **|||||||||||||||||||||||||||| |||||**  **Sbjct: 617978 tgtctgaatctgagtcgctatctgagtctgaatc 617945**  **Score = 60.0 bits (30), Expect = 6e-11**  **Identities = 33/34 (97%)**  **Strand = Plus / Minus**  **:**  **Query: 1 tgtctgaatctgagtcgctatctgagtcggaatc 34**  **|||||||||||||||||||||||||||| |||||**  **Sbjct: 618158 tgtctgaatctgagtcgctatctgagtctgaatc 618125**  **Database: NC_007790.1.2.3_DB.fna**  **Posted date: Jan 3, 2010 9:09 PM**  **Number of letters in database: 2,917,469**  **Number of sequences in database: 4** | 5 copies: 3 with “A”, 1 with “C” and the 1 with “G”. |
| 622075 | sdrE protein | T:10 C:219 | **>gi|87159884|ref|NC_007793.1| Staphylococcus aureus subsp. aureus  USA300_FPR3757, complete genome  Length = 2872769   Score = 69.9 bits (35), Expect = 6e-14  Identities = 35/35 (100%)  Strand = Plus / Plus   Query: 1 tcagatagcgactcagactcagatagcgactcaga 35  ||||||||||||||||||||||||||||||||||| Sbjct: 613658 tcagatagcgactcagactcagatagcgactcaga 613692   Score = 69.9 bits (35), Expect = 6e-14  Identities = 35/35 (100%)  Strand = Plus / Plus   Query: 1 tcagatagcgactcagactcagatagcgactcaga 35  ||||||||||||||||||||||||||||||||||| Sbjct: 613676 tcagatagcgactcagactcagatagcgactcaga 613710   Score = 63.9 bits (32), Expect = 4e-12  Identities = 32/32 (100%)  Strand = Plus / Plus   Query: 4 gatagcgactcagactcagatagcgactcaga 35  |||||||||||||||||||||||||||||||| Sbjct: 613457 gatagcgactcagactcagatagcgactcaga 613488   Score = 61.9 bits (31), Expect = 1e-11  Identities = 34/35 (97%)   Query: 1 tcagatagcgactcagactcagatagcgactcaga 35  ||||||||||||||||||||||||||||| ||||| Sbjct: 618224 tcagatagcgactcagactcagatagcgattcaga 618258   Score = 61.9 bits (31), Expect = 1e-11  Identities = 34/35 (97%)  Strand = Plus / Plus   Query: 1 tcagatagcgactcagactcagatagcgactcaga 35  ||||| ||||||||||||||||||||||||||||| Sbjct: 622070 tcagacagcgactcagactcagatagcgactcaga 622104   Score = 61.9 bits (31), Expect = 1e-11  Identities = 34/35 (97%)  Strand = Plus / Minus   Query: 1 tcagatagcgactcagactcagatagcgactcaga 35  ||||| ||||||||||||||||||||||||||||| Sbjct: 2774870 tcagacagcgactcagactcagatagcgactcaga 2774836    Database: NC_007790.1.2.3_DB.fna  Posted date: Jan 3, 2010 9:09 PM  Number of letters in database: 2,917,469  Number of sequences in database: 4** | 6 copies: 2 with “C” and 4 with “T”. |
| 861520 | clumping factor A | A:5 G:74 | **>gi|87159884|ref|NC_007793.1| Staphylococcus aureus subsp. aureus**  **USA300_FPR3757, complete genome**  **Length = 2872769**  **Score = 69.9 bits (35), Expect = 6e-14**  **Identities = 35/35 (100%)**  **Strand = Plus / Minus**    **Query: 1 gactcggattcagatagcgactcagactcagatag 35**  **|||||||||||||||||||||||||||||||||||**  **Sbjct: 2774609 gactcggattcagatagcgactcagactcagatag 2774575**  **Score = 61.9 bits (31), Expect = 1e-11**  **Identities = 34/35 (97%)**  **Strand = Plus / Plus**    **Query: 1 gactcggattcagatagcgactcagactcagatag 35**  **||||| |||||||||||||||||||||||||||||**  **Sbjct: 613649 gactcagattcagatagcgactcagactcagatag 613683**  **Score = 61.9 bits (31), Expect = 1e-11**  **Identities = 34/35 (97%)**  **Strand = Plus / Plus**    **Query: 1 gactcggattcagatagcgactcagactcagatag 35**  **||||||||||||||||||||||||||||| |||||**  **Sbjct: 861491 gactcggattcagatagcgactcagactcggatag 861525**  **Database: NC_007790.1.2.3_DB.fna**  **Posted date: Jan 3, 2010 9:09 PM**  **Number of letters in database: 2,917,469**  **Number of sequences in database: 4** | 3 copies: 2 with “G” and 1 with “A”. |
| 2295214 | 23S ribosomal RNA | A:10 G:148 | **>gi|87159884|ref|NC_007793.1| Staphylococcus aureus subsp. aureus  USA300_FPR3757, complete genome  Length = 2872769   Score = 73.8 bits (37), Expect = 4e-15  Identities = 37/37 (100%)  Strand = Plus / Minus   Query: 1 cttcccatttcgctcgccgctactaagggaatcgaat 37  ||||||||||||||||||||||||||||||||||||| Sbjct: 515075 cttcccatttcgctcgccgctactaagggaatcgaat 515039   Score = 65.9 bits (33), Expect = 1e-12  Identities = 36/37 (97%)  Strand = Plus / Minus   Query: 1 cttcccatttcgctcgccgctactaagggaatcgaat 37  |||||| |||||||||||||||||||||||||||||| Sbjct: 558678 cttcccgtttcgctcgccgctactaagggaatcgaat 558642   Score = 65.9 bits (33), Expect = 1e-12  Identities = 36/37 (97%)  Strand = Plus / Plus   Query: 1 cttcccatttcgctcgccgctactaagggaatcgaat 37  |||||| |||||||||||||||||||||||||||||| Sbjct: 2000430 cttcccgtttcgctcgccgctactaagggaatcgaat 2000466   Score = 65.9 bits (33), Expect = 1e-12  Identities = 36/37 (97%)  Strand = Plus / Plus   Query: 1 cttcccatttcgctcgccgctactaagggaatcgaat 37  |||||| |||||||||||||||||||||||||||||| Sbjct: 2178654 cttcccgtttcgctcgccgctactaagggaatcgaat 2178690   Score = 65.9 bits (33), Expect = 1e-12  Identities = 36/37 (97%)  Strand = Plus / Plus  Query: 1 cttcccatttcgctcgccgctactaagggaatcgaat 37  |||||| |||||||||||||||||||||||||||||| Sbjct: 2295208 cttcccgtttcgctcgccgctactaagggaatcgaat 2295244   Database: NC_007790.1.2.3_DB.fna  Posted date: Jan 3, 2010 9:09 PM  Number of letters in database: 2,917,469  Number of sequences in database: 4** | 5 copies: 4 with “G” and 1 with “A”. |

Note: query sequences are from SRX007711 that show heterogeneity.
