# Supplementary material for: GenHtr: a tool for comparative assessment of genetic heterogeneity in microbial genomes generated by massive short-read sequencing
Source: BMC Bioinformatics. 2010 Oct 12;11:508. doi: 10.1186/1471-2105-11-508 (PMC2967562; doi:10.1186/1471-2105-11-508)
Supplement: Additional file 3 — Table S3: Distribution of "heterogeneity" sites at the isogenic reference genome. [file 1471-2105-11-508-S3.DOC]

**Additional file 3** **Table S3**. Distribution of “heterogeneity” sites at the isogenic reference genome

| **Number of Heterogeneity sites** | **Function Category** | **Percentage** |
| --- | --- | --- |
| 1 | Serine protease splb | 0.001019368 |
| 1 | Alcohol dehydrogenase, zinc-containing | 0.001019368 |
| 1 | Serine protease splc | 0.001019368 |
| 1 | Superantigen-like protein 7 | 0.001019368 |
| 1 | Lantibiotic epidermin biosynthesis protein epia | 0.001019368 |
| 1 | Panton-Valentine leukocidin, luks-PV | 0.001019368 |
| 1 | Phislt ORF 82-like protein | 0.001019368 |
| 1 | Putative alcohol dehydrogenase | 0.001019368 |
| 1 | Phislt ORF53-like protein | 0.001019368 |
| 1 | Ser trna | 0.001019368 |
| 2 | L-lactate permease | 0.002038736 |
| 2 | 2-C-methyl-D-erythritol 4-phosphate cytidylyltransferase | 0.002038736 |
| 2 | His trna | 0.002038736 |
| 2 | Phi77 ORF069-like protein | 0.002038736 |
| 2 | Phislt ORF484-like protein, lysin | 0.002038736 |
| 2 | Autolysin | 0.002038736 |
| 2 | Arginine deiminase | 0.002038736 |
| 2 | Phipvl ORF057-like protein, transcriptional activator rinb | 0.002038736 |
| 2 | Phislt ORF 81b-like protein | 0.002038736 |
| 2 | Gamma-hemolysin component B | 0.002038736 |
| 2 | Phage terminase family protein | 0.002038736 |
| 2 | Phipvl ORF052-like protein | 0.002038736 |
| 2 | Phi77 ORF031-like protein | 0.002038736 |
| 2 | Phislt ORF 50-like protein | 0.002038736 |
| 2 | Truncated amidase | 0.002038736 |
| 3 | Serine protease sple | 0.003058104 |
| 3 | Phipvl ORF051-like protein | 0.003058104 |
| 3 | Phislt ORF80-like protein | 0.003058104 |
| 3 | Phislt ORF78-like protein | 0.003058104 |
| 4 | Putative restriction/modification system specificity protein | 0.004077472 |
| 4 | Type I restriction-modification enzyme, S subunit | 0.004077472 |
| 4 | Integrase/recombinase | 0.004077472 |
| 4 | CHAP domain-contain protein | 0.004077472 |
| 4 | Leukotoxin lukd | 0.004077472 |
| 4 | IS1181, transposase | 0.004077472 |
| 4 | IS1272, transposase | 0.004077472 |
| 4 | Phislt ORF153-like protein | 0.004077472 |
| 5 | Immunoglobulin G binding protein A precursor | 0.00509684 |
| 6 | Panton-Valentine leukocidin, lukf-PV | 0.006116208 |
| 7 | Superantigen-like protein | 0.007135576 |
| 8 | IS200 family transposase | 0.008154944 |
| 8 | Putative staphylococcal tandem lipoprotein | 0.008154944 |
| 9 | Putative teichoic acid biosynthesis protein B | 0.009174312 |
| 9 | Putative teichoic acid biosynthesis protein | 0.009174312 |
| 10 | Serine protease splf | 0.01019368 |
| 10 | Serine protease spld | 0.01019368 |
| 12 | Clumping factor A | 0.012232416 |
| 12 | Cell surface protein | 0.012232416 |
| 12 | Deoxyribose-phosphate aldolase | 0.012232416 |
| 13 | Transposase, truncation | 0.013251784 |
| 17 | Fibronectin binding protein B | 0.017329256 |
| 18 | Putative lipoprotein | 0.018348624 |
| 19 | Fibronectin binding protein A | 0.019367992 |
| 20 | 16S ribosomal RNA | 0.02038736 |
| 20 | Type I restriction-modification system, M subunit | 0.02038736 |
| 24 | Transposase | 0.024464832 |
| 25 | 23S ribosomal RNA | 0.0254842 |
| 44 | Putative transposase | 0.044852192 |
| 56 | SdrE protein | 0.057084608 |
| 59 | Clumping factor B | 0.060142712 |
| 60 | SdrC protein | 0.06116208 |
| 75 | SdrD protein | 0.076452599 |
| 127 | Staphylococcal tandem lipoprotein | 0.129459735 |
| 212 | Hypothetical protein | 0.216106014 |
