# Supplementary material for: GenHtr: a tool for comparative assessment of genetic heterogeneity in microbial genomes generated by massive short-read sequencing
Source: BMC Bioinformatics. 2010 Oct 12;11:508. doi: 10.1186/1471-2105-11-508 (PMC2967562; doi:10.1186/1471-2105-11-508)
Supplement: Additional file 4 — Table S4: Affect of read depth on the performance of Maq in discovering SNPs. [file 1471-2105-11-508-S4.DOC]

**Additional file 4 Table S4.** Affect of read depth on the performance of Maq in discovering SNPs.

| **Data** | **Maq Paramters^a^** | | | | | | | | | | **SNP?** |
| --- | --- | --- | --- | --- | --- | --- | --- | --- | --- | --- | --- |
|  | **RB** | **CB** | **CQ** | **RD** | **NH** | **HQ** | **MQ** | **SBC** | **S/T** | **TBC** |  |
| Solexa Reads covering SNP at the position of 395176 | | | | | | | | | | | |
| gi|87159884|ref|NC_007793.1| | A | T | 141 | 38 | 1 | 58 | 62 | N | 255 | N | Yes |
| Simulated data with read depth of 4 | | | | | | | | | | | |
| 395176.Solexa.4.1000.0 | A | T | 39 | 4 | 1 | 36 | 38 | N | 86 | N | No |
| 395176.Solexa.4.1000.1 | A | T | 39 | 4 | 1 | 36 | 38 | N | 35 | N | No |
| 395176.Solexa.4.1000.2 | A | T | 39 | 4 | 1 | 36 | 38 | N | 44 | N | No |
| 395176.Solexa.4.1000.3 | A | T | 39 | 4 | 1 | 36 | 38 | N | 69 | N | No |
| 395176.Solexa.4.1000.4 | A | T | 39 | 4 | 1 | 36 | 38 | N | 60 | N | No |
| 395176.Solexa.4.1000.5 | C | A | 27 | 1 | 1 | 27 | 0 | N | 3 | N | No |
| 395176.Solexa.4.1000.6 | A | T | 39 | 4 | 1 | 36 | 38 | N | 42 | N | No |
| 395176.Solexa.4.1000.7 | A | T | 39 | 4 | 1 | 36 | 38 | N | 26 | N | No |
| 395176.Solexa.4.1000.8 | A | T | 39 | 4 | 1 | 27 | 38 | N | 20 | N | No |
| 395176.Solexa.4.1000.9 | A | T | 39 | 4 | 1 | 36 | 38 | N | 34 | N | No |
| 395176.Solexa.4.1000.10 | A | T | 39 | 4 | 1 | 36 | 38 | N | 55 | N | No |
| Simulated data with read depth of 6 | | | | | | | | | | | |
| 395176.Solexa.6.1000.0 | A | T | 45 | 6 | 1 | 38 | 44 | N | 49 | N | No |
| 395176.Solexa.6.1000.1 | A | T | 45 | 6 | 1 | 38 | 44 | N | 96 | N | No |
| 395176.Solexa.6.1000.2 | C | A | 29 | 1 | 1 | 29 | 0 | N | 1 | N | No |
| 395176.Solexa.6.1000.3 | A | T | 45 | 6 | 1 | 38 | 44 | N | 75 | N | No |
| 395176.Solexa.6.1000.4 | A | T | 45 | 6 | 1 | 38 | 44 | N | 94 | N | No |
| 395176.Solexa.6.1000.5 | A | T | 45 | 6 | 1 | 38 | 44 | N | 53 | N | No |
| 395176.Solexa.6.1000.6 | A | T | 45 | 6 | 1 | 38 | 44 | N | 82 | N | No |
| 395176.Solexa.6.1000.7 | A | T | 45 | 6 | 1 | 38 | 44 | N | 62 | N | No |
| 395176.Solexa.6.1000.8 | A | T | 45 | 6 | 1 | 38 | 44 | N | 79 | N | No |
| 395176.Solexa.6.1000.9 | A | T | 45 | 6 | 1 | 38 | 44 | N | 67 | N | No |
| 395176.Solexa.6.1000.10 | A | T | 45 | 6 | 1 | 38 | 44 | N | 79 | N | No |
| Simulated data with read depth of 8 | | | | | | | | | | | |
| 395176.Solexa.8.1000.0 | A | T | 51 | 8 | 1 | 39 | 50 | N | 91 | N | No |
| 395176.Solexa.8.1000.1 | A | T | 51 | 8 | 1 | 39 | 50 | N | 57 | N | No |
| 395176.Solexa.8.1000.2 | A | T | 51 | 8 | 1 | 39 | 50 | N | 97 | N | No |
| 395176.Solexa.8.1000.3 | A | T | 51 | 8 | 1 | 36 | 50 | N | 78 | N | No |
| 395176.Solexa.8.1000.4 | A | T | 51 | 8 | 1 | 39 | 50 | N | 54 | N | No |
| 395176.Solexa.8.1000.5 | A | T | 51 | 8 | 1 | 39 | 50 | N | 126 | N | No |
| 395176.Solexa.8.1000.6 | A | T | 51 | 8 | 1 | 39 | 50 | N | 121 | N | No |
| 395176.Solexa.8.1000.7 | A | T | 51 | 8 | 1 | 39 | 50 | N | 84 | N | No |
| 395176.Solexa.8.1000.8 | C | A | 30 | 1 | 1 | 30 | 0 | N | 0 | N | No |
| 395176.Solexa.8.1000.9 | A | T | 51 | 8 | 1 | 39 | 50 | N | 93 | N | No |
| 395176.Solexa.8.1000.10 | A | T | 51 | 8 | 1 | 39 | 50 | N | 102 | N | No |
| Simulated data with read depth of 10 | | | | | | | | | | | |
| 395176.Solexa.10.1000.0 | A | T | 57 | 10 | 1 | 40 | 56 | N | 87 | N | Yes |
| 395176.Solexa.10.1000.1 | A | T | 57 | 10 | 1 | 40 | 56 | N | 124 | N | Yes |
| 395176.Solexa.10.1000.2 | A | T | 57 | 10 | 1 | 40 | 56 | N | 125 | N | Yes |
| 395176.Solexa.10.1000.3 | A | T | 57 | 10 | 1 | 40 | 56 | N | 89 | N | Yes |
| 395176.Solexa.10.1000.4 | A | T | 57 | 10 | 1 | 40 | 56 | N | 149 | N | Yes |
| 395176.Solexa.10.1000.5 | A | T | 57 | 10 | 1 | 40 | 56 | N | 94 | N | Yes |
| 395176.Solexa.10.1000.6 | A | T | 57 | 10 | 1 | 40 | 56 | N | 117 | N | Yes |
| 395176.Solexa.10.1000.7 | A | T | 57 | 10 | 1 | 40 | 56 | N | 113 | N | Yes |
| 395176.Solexa.10.1000.8 | A | T | 57 | 10 | 1 | 40 | 56 | N | 113 | N | Yes |
| 395176.Solexa.10.1000.9 | A | T | 57 | 10 | 1 | 40 | 56 | N | 148 | N | Yes |
| 395176.Solexa.10.1000.10 | A | T | 57 | 10 | 1 | 40 | 56 | N | 117 | N | Yes |

Note: a. **RB:** reference base, **CB:** consensus base, **CQ:** Phred-like consensus quality, **RD:** read depth, **NH:** the average number of hits of reads covering this position, **HQ:** the highest mapping quality of the reads covering the position, **MQ:** the minimum consensus quality in the 3bp flanking regions at each side of the site (6bp in total), **SBC**: the second best call, **S/T**: log likelihood ratio of the second best and the third best call, **TBC**: and the third best call.
