# Supplementary material for: GenHtr: a tool for comparative assessment of genetic heterogeneity in microbial genomes generated by massive short-read sequencing
Source: BMC Bioinformatics. 2010 Oct 12;11:508. doi: 10.1186/1471-2105-11-508 (PMC2967562; doi:10.1186/1471-2105-11-508)
Supplement: Additional file 5 — Table S5: Heterogeneity and SNPs detected by Maq analysis. [file 1471-2105-11-508-S5.DOC]

**Additional file 5 Table S5.** Heterogeneity and SNPs detected by Maq analysis

| **Data** | **Maq Paramters^a^** | | | | | | | | | | **Heterogeneity?** |
| --- | --- | --- | --- | --- | --- | --- | --- | --- | --- | --- | --- |
|  | **RB** | **CB** | **CQ** | **RD** | **NH** | **HQ** | **MQ** | **SBC** | **S/T** | **TBC** |  |
| 36015 | C | Y | 255 | 255 | 1 | 63 | 62 | C | 255 | T | Yes |
| 36087 | A | R | 255 | 255 | 1.25 | 63 | 62 | G | 130 | A | Yes |
| 36269 | C | Y | 255 | 255 | 1.31 | 63 | 62 | T | 57 | C | Yes |
| 36315 | G | R | 255 | 255 | 1.81 | 63 | 62 | G | 0 | A | Yes |
| 36378 | C | Y | 255 | 255 | 1.06 | 63 | 62 | C | 129 | T | Yes |
| 36581 | C | Y | 255 | 255 | 1.25 | 63 | 62 | T | 179 | C | Yes |
