# Supplementary material for: GenHtr: a tool for comparative assessment of genetic heterogeneity in microbial genomes generated by massive short-read sequencing
Source: BMC Bioinformatics. 2010 Oct 12;11:508. doi: 10.1186/1471-2105-11-508 (PMC2967562; doi:10.1186/1471-2105-11-508)
Supplement: Additional file 6 — Table S6: Genotype analysis by GenHtr [file 1471-2105-11-508-S6.DOC]

***Additional file 6*** ***Table S6.*** *Genotype analysis by Gen^Htr^*

| **Chrom Position** | **Genotype**  **at**  **SRX007711** | **Genotype**  **at**  **FPR3757** | **Read Depth** | **Average Phred Values** | **Highest Phred Values** | **Mean Probability to Be Incorrect Reads** | **Function Description** |
| --- | --- | --- | --- | --- | --- | --- | --- |
| 36015 | T:12 C:328 | T:10 C:72 | 12 | 12.33333333 | 40 | 0.058434141 | putative transposase |
| 36087 | A:401 G:106 | A:126 G:20 | 106 | 14.10377358 | 40 | 0.038870725 | putative transposase |
| 36269 | A:1 T:95 C:451 | T:10 C:166 | 96 | 16.4375 | 40 | 0.022711719 | putative transposase |
| 36315 | A:137 T:2 G:523 | A:10 G:185 | 139 | 19.17266187 | 40 | 0.012098564 | putative transposase |
| 36378 | T:283 C:174 | T:40 C:74 | 283 | 20.72438163 | 40 | 0.008463731 | putative transposase |
| 36581 | A:1 T:103 C:446 | T:5 C:161 | 104 | 20.84615385 | 40 | 0.008229712 | Intergenic |
