# Supplementary material for: GenHtr: a tool for comparative assessment of genetic heterogeneity in microbial genomes generated by massive short-read sequencing
Source: BMC Bioinformatics. 2010 Oct 12;11:508. doi: 10.1186/1471-2105-11-508 (PMC2967562; doi:10.1186/1471-2105-11-508)
Supplement: Additional file 7 — Table S7: Mutations detected in the S. aureus JH1 and JH9 by simulation [file 1471-2105-11-508-S7.DOC]

**Additional file7 Table S7.** Mutations detected in the *S. aureus* JH1 and JH9 by simulation

| **Chrom Position** | **Gene position** | **Genotype in Heterogeneity** | | | | **Functional Description** |
| --- | --- | --- | --- | --- | --- | --- |
|  |  | **JH1** | **Number of Substitutions** | **JH9** | **Number of Substitution** |  |
| 543690 | 280 | A:76 C:204 | 2/6 | C:306 | 0/6 | 23S ribosomal RNA |
| 543950 | 540 | A:38 G:255 | 1/6 | G:306 | 0/6 | 23S ribosomal RNA |
| 587981 | 280 | A:76 C:204 | 3/6 | C:306 | 0/6 | 23S ribosomal RNA |
| 589285 | 1584 | A:153 T:114 | 3/6 | A:102 T:152 | 2/6 | 23S ribosomal RNA |
| 593419 | 540 | A:38 G:255 | 1/6 | G:306 | 0/6 | 23S ribosomal RNA |
| 594463 | 1584 | A:153 T:114 | 3/6 | A:102 T:152 | 2/6 | 23S ribosomal RNA |
| 2044885 | 280 | T:38 C:255 | 1/6 | C:306 | 0/6 | 23S ribosomal RNA |
| 2045145 | 540 | T:76 G:204 | 2/6 | G:306 | 0/6 | 23S ribosomal RNA |
| 2217835 | 280 | T:38 C:255 | 1/6 | C:306 | 0/6 | 23S ribosomal RNA |
| 2218095 | 540 | T:76 G:204 | 2/6 | G:306 | 0/6 | 23S ribosomal RNA |
| 2332922 | 280 | T:38 C:255 | 1/6 | C:306 | 0/6 | 23S ribosomal RNA |
| 2333182 | 540 | T:76 G:204 | 2/6 | G:306 | 0/6 | 23S ribosomal RNA |
