# Supplementary figures and images for: GenHtr: a tool for comparative assessment of genetic heterogeneity in microbial genomes generated by massive short-read sequencing
Source: BMC Bioinformatics. 2010 Oct 12;11:508. doi: 10.1186/1471-2105-11-508 (PMC2967562; doi:10.1186/1471-2105-11-508)

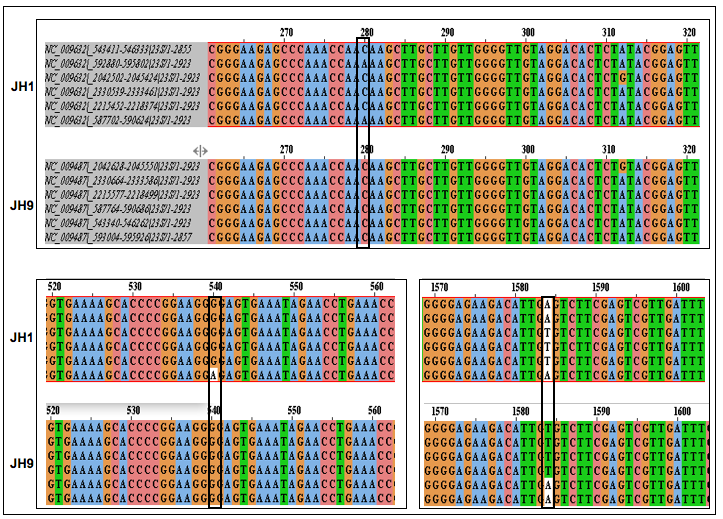


**Additional file 8 Figure S1.** Differences in mutations of 23S ribosomal RNA between JH1 and JH9.

Supplement: Additional file 8 — Fig. S1: Differences in mutations of 23 S ribosomal RNA between JH1 and JH9. [file 1471-2105-11-508-S8.DOC]
