# Supplementary material for: GenHtr: a tool for comparative assessment of genetic heterogeneity in microbial genomes generated by massive short-read sequencing
Source: BMC Bioinformatics. 2010 Oct 12;11:508. doi: 10.1186/1471-2105-11-508 (PMC2967562; doi:10.1186/1471-2105-11-508)
Supplement: Additional file 9 — Table S8: Blat Alignment differences between two different models of analysis [file 1471-2105-11-508-S9.DOC]

**Additional file 9 Table S8.** Blat Alignment differences between two different models of analysis

| **Sample ID** | **Blat Alignment with RGDF as Queries against Solexa Reads** | **Blat Alignment with Solexa Reads as Queries against Genome faToTwo DB** |
| --- | --- | --- |
| **2B1_11600** | >SRR022865_202_2  Length = 37  Score = 67 bits (173), Expect = 2e-11  Identities = 36/37 (97%)  Strand = Plus / Plus  **Query: 393 gctatatcgcacagcagatttagacatttcttttaaa 429**  **||||||||||||||||||||||||||||| |||||||**  **Sbjct: 1 gctatatcgcacagcagatttagacatttattttaaa 37** | > >gi|87159884|ref|NC_007793.1|  Length = 2872769  Score = 56 bits (145), Expect = 4e-08  Identities = 29/29 (100%)  Strand = Plus / Plus  **Query: 1 gctatatcgcacagcagatttagacattt 29**  **|||||||||||||||||||||||||||||**  **Sbjct: 1829393 gctatatcgcacagcagatttagacattt 1829421**  Score = 15 bits (39), Expect = 1e+05  Identities = 8/8 (100%)  Strand = Plus / Plus  **Query: 30 attttaaa 37**  **||||||||**  **Sbjct: 1829821 attttaaa 1829828** |
| **SRR022865_550_2** | >SRR022865_550_2  Length = 37  Score = 69 bits (178), Expect = 5e-12  Identities = 36/37 (97%)  Strand = Plus / Plus  **Query: 622 tctgtcatttcgttcttagtagtaggtttcgtgcctt 658**  **|||||||||||||||||||||||||||||||| ||||**  **Sbjct: 1 tctgtcatttcgttcttagtagtaggtttcgtacctt 37** | >gi|87159884|ref|NC_007793.1|  Length = 2872769  Score = 62 bits (160), Expect = 7e-10  Identities = 32/32 (100%)  Strand = Plus / Plus  **Query: 1 tctgtcatttcgttcttagtagtaggtttcgt 32**  **||||||||||||||||||||||||||||||||**  **Sbjct: 626622 tctgtcatttcgttcttagtagtaggtttcgt 626653**  Score = 10 bits (25), Expect = 4e+06  Identities = 5/5 (100%)  Strand = Plus / Plus  **Query: 33 acctt 37**  **|||||**  **Sbjct: 626693 acctt 626697** |
| **>SRR022865_801_2** | >SRR022865_801_2  Length = 37  Score = 65 bits (168), Expect = 8e-11  Identities = 35/36 (97%)  Strand = Plus / Plus  **Query: 290 cgttgataatgattgttgataaggtttgattgccat 325**  **||||||||||||||||||||||||||||||| ||||**  **Sbjct: 1 cgttgataatgattgttgataaggtttgatttccat 36** | > >gi|87159884|ref|NC_007793.1|  Length = 2872769  Score = 59 bits (154), Expect = 4e-09  Identities = 31/31 (100%)  Strand = Plus / Plus  **Query: 1 cgttgataatgattgttgataaggtttgatt 31**  **|||||||||||||||||||||||||||||||**  **Sbjct: 1243290 cgttgataatgattgttgataaggtttgatt 1243320**  Score = 12 bits (30), Expect = 9e+05  Identities = 6/6 (100%)  Strand = Plus / Plus  **Query: 32 tccatc 37**  **||||||**  **Sbjct: 1243791 tccatc 1243796** |
| **SRR022865_825_2** | >SRR022865_825_2  Length = 37  Score = 68 bits (176), Expect = 8e-12  Identities = 36/37 (97%)  Strand = Plus / Plus  **Query: 328 aaacatcattggcttaatcaacattcaattcgttata 364**  **|||||||||||||||||||||||||||||||| ||||**  **Sbjct: 1 aaacatcattggcttaatcaacattcaattcgctata 37** | >gi|87159884|ref|NC_007793.1|  Length = 2872769  Score = 59 bits (154), Expect = 4e-09  Identities = 31/31 (100%)  Strand = Plus / Plus  **Query: 1 aaacatcattggcttaatcaacattcaattc 31**  **|||||||||||||||||||||||||||||||**  **Sbjct: 1201328 aaacatcattggcttaatcaacattcaattc 1201358**  Score = 12 bits (30), Expect = 1e+06  Identities = 6/6 (100%)  Strand = Plus / Plus  **Query: 32 gctata 37**  **||||||**  **Sbjct: 1201777 gctata 1201782** |
| **SRR022865_1327_2** | >SRR022865_1327_2  Length = 37  Score = 67 bits (172), Expect = 3e-11  Identities = 36/37 (97%)  Strand = Plus / Plus  **Query: 525 gcttgatgttgttgactatttaaaaatgcttgttcaa 561**  **||||||||||||||||||||||||||||||| |||||**  **Sbjct: 1 gcttgatgttgttgactatttaaaaatgctttttcaa 37** | >gi|87159884|ref|NC_007793.1|  Length = 2872769  Score = 59 bits (154), Expect = 4e-09  Identities = 31/31 (100%)  Strand = Plus / Plus  **Query: 1 gcttgatgttgttgactatttaaaaatgctt 31**  **|||||||||||||||||||||||||||||||**  **Sbjct: 1497525 gcttgatgttgttgactatttaaaaatgctt 1497555**  Score = 11 bits (29), Expect = 1e+06  Identities = 6/6 (100%)  Strand = Plus / Plus  **Query: 32 tttcaa 37**  **||||||**  **Sbjct: 1497799 tttcaa 1497804** |
