# Supplementary material for: GenHtr: a tool for comparative assessment of genetic heterogeneity in microbial genomes generated by massive short-read sequencing
Source: BMC Bioinformatics. 2010 Oct 12;11:508. doi: 10.1186/1471-2105-11-508 (PMC2967562; doi:10.1186/1471-2105-11-508)
Supplement: Additional file 10 — Table S9: Percentile of Phred values on 37 positions on 5000 Solexa reads. [file 1471-2105-11-508-S10.DOC]

**Additional file 10 Table S9.** Percentile of Phred values on 37 positions on 5000 Solexa reads.

| **Sequence Position** |  | **Percentile (from high to low)** | | | | | | |
| --- | --- | --- | --- | --- | --- | --- | --- | --- |
|  | **Mean** | **5%** | **10%** | **25%** | **50%** | **75%** | **90%** | **95%** |
| 1 | 34 | 40 | 40 | 40 | 40 | 34 | **19** | **12** |
| 2 | 28 | 40 | 40 | 40 | 32 | 20 | **10** | **6** |
| 3 | 31 | 40 | 40 | 40 | 39 | 23 | **12** | **8** |
| 4 | 30 | 40 | 40 | 40 | 38 | 22 | **11** | **7** |
| 5 | 30 | 40 | 40 | 40 | 37 | 22 | **12** | **7** |
| 6 | 28 | 40 | 40 | 40 | 32 | 19 | **10** | **6** |
| 7 | 27 | 40 | 40 | 40 | 29 | 18 | **10** | **5** |
| 8 | 26 | 40 | 40 | 40 | 28 | 17 | **8** | **5** |
| 9 | 26 | 40 | 40 | 40 | 27 | 16 | **9** | **5** |
| 10 | 26 | 40 | 40 | 40 | 27 | 17 | **9** | **5** |
| 11 | 24 | 40 | 40 | 39 | 24 | 14 | **8** | **5** |
| 12 | 24 | 40 | 40 | 39 | 24 | 13 | **8** | **5** |
| 13 | 22 | 40 | 40 | 34 | 22 | 13 | **7** | **5** |
| 14 | 21 | 40 | 40 | 32 | 19 | **10** | **5** | **4** |
| 15 | 20 | 40 | 40 | 29 | 18 | **10** | **6** | **4** |
| 16 | 19 | 40 | 40 | 28 | 18 | **10** | **6** | **4** |
| 17 | 18 | 40 | 40 | 26 | 16 | **10** | **5** | **4** |
| 18 | 17 | 40 | 40 | 23 | 14 | **9** | **5** | **3** |
| 19 | 16 | 40 | 37 | 22 | 13 | **8** | **5** | **3** |
| 20 | 14 | 40 | 34 | 20 | **11** | **7** | **4** | **3** |
| 21 | 15 | 40 | 36 | 20 | **11** | **7** | **4** | **3** |
| 22 | 13 | 40 | 33 | 18 | **10** | **6** | **4** | **2** |
| 23 | 13 | 40 | 29 | 17 | **10** | **6** | **4** | **3** |
| 24 | 12 | 40 | 29 | 16 | **10** | **6** | **3** | **2** |
| 25 | 12 | 40 | 27 | 14 | **10** | **5** | **3** | **2** |
| 26 | 12 | 40 | 27 | 14 | **9** | **5** | **3** | **2** |
| 27 | 10 | 40 | 24 | 13 | **8** | **5** | **3** | **1** |
| 28 | 11 | 40 | 24 | 13 | **8** | **5** | **3** | **1** |
| 29 | 10 | 40 | 23 | **12** | **8** | **5** | **3** | **1** |
| 30 | 10 | 40 | 23 | **12** | **8** | **4** | **3** | **1** |
| 31 | 9 | 39 | 20 | **11** | **7** | **4** | **2** | **1** |
| 32 | 9 | 39 | 20 | **10** | **6** | **4** | **2** | **1** |
| 33 | 8 | 36 | 19 | **10** | **6** | **4** | **2** | **1** |
| 34 | 8 | 34 | 18 | **10** | **5** | **4** | **2** | **1** |
| 35 | 8 | 31 | 18 | **10** | **5** | **3** | **2** | **1** |
| 36 | 8 | 31 | 17 | **10** | **5** | **3** | **1** | **1** |
| 37 | 8 | 34 | 18 | **10** | **5** | **3** | **1** | **1** |

Note: the bolded cells represent Phred values with a converted probability of greater than 0.05 (to be incorrect base calls).
