# Supplementary material for: GenHtr: a tool for comparative assessment of genetic heterogeneity in microbial genomes generated by massive short-read sequencing
Source: BMC Bioinformatics. 2010 Oct 12;11:508. doi: 10.1186/1471-2105-11-508 (PMC2967562; doi:10.1186/1471-2105-11-508)
Supplement: Additional file 11 — Table S10: A list of positions that are share same genotypes between the reference genome and those from the newly sequenced S. aureus subsp. USA300 cell line. [file 1471-2105-11-508-S11.DOC]

**Additional file 11 Table S10.** A list of positions that are share same genotypes between the reference genome and those from the newly sequenced *S. aureus* subsp. USA300 cell line. Base calls at all these positions have insignificant Phred values

| **Chrom Position** | **Loci**  **at**  **SRX007711** | **Loci**  **at**  **FPR3757** | **Count of Nucleotides in “Minority” forms** | **Phred Values** | | **Mean Probability Incorrect base calls** | **Phred Values for the First Ten Reads** | | | | | | | | | |
| --- | --- | --- | --- | --- | --- | --- | --- | --- | --- | --- | --- | --- | --- | --- | --- | --- |
|  |  |  |  | **Mean** | **Max** |  |  |  |  |  |  |  |  |  |  |  |
| 515236 | A:11 G:504 | A:10 G:148 | 11 | 6.18 | 14 | 0.24 | 18 | 4 | 9 | 5 | 14 | 3 | 3 | 11 | 10 | 4 |
| 2629224 | T:159 C:16 | T:37 C:5 | 16 | 6.12 | 11 | 0.24 | 1 | 8 | 8 | 6 | 5 | 3 | 7 | 7 | 6 | 5 |
| 2070948 | A:10 T:3 G:468 | A:10 G:196 | 13 | 6 | 25 | 0.25 | 3 | 25 | 3 | 4 | 3 | 10 | 5 | 3 | 3 | 5 |
| 302707 | A:9 G:115 | A:5 G:37 | 9 | 6 | 21 | 0.25 | 5 | 5 | 4 | 3 | 5 | 2 | 21 | 6 | 3 |  |
| 1910250 | A:3 T:10 C:468 | T:10 C:196 | 13 | 6 | 25 | 0.25 | 3 | 25 | 3 | 4 | 3 | 10 | 5 | 3 | 3 | 5 |
| 621805 | T:2 C:535 | T:2 C:185 | 2 | 6 | 9 | 0.25 | 3 | 9 |  |  |  |  |  |  |  |  |
| 861160 | A:14 C:78 | A:5 C:42 | 14 | 5.92 | 10 | 0.25 | 5 | 8 | 5 | 5 | 8 | 6 | 8 | 15 | 5 | 5 |
| 1412990 | A:207 G:6 | A:83 G:5 | 6 | 5.83 | 10 | 0.26 | 5 | 6 | 3 | 10 | 7 | 4 |  |  |  |  |
| 159424 | A:10 G:111 | A:5 G:37 | 10 | 5.8 | 30 | 0.26 | 2 | 30 | 5 | 5 | 5 | 3 | 3 | 1 | 3 | 1 |
| 1154622 | A:214 T:1 G:3 | A:128 G:5 | 4 | 5.75 | 8 | 0.26 | 4 | 5 | 6 | 8 |  |  |  |  |  |  |
| 1443678 | A:4 G:239 | A:5 G:74 | 4 | 5.75 | 9 | 0.26 | 9 | 5 | 5 | 4 |  |  |  |  |  |  |
| 1917160 | T:4 C:239 | T:5 C:74 | 4 | 5.75 | 9 | 0.26 | 9 | 5 | 5 | 4 |  |  |  |  |  |  |
| 834724 | A:7 T:340 | A:5 T:197 | 7 | 5.71 | 9 | 0.26 | 2 | 5 | 9 | 5 | 7 | 7 | 5 |  |  |  |
| 2031041 | T:145 C:7 | T:200 C:5 | 7 | 5.71 | 7 | 0.26 | 5 | 7 | 5 | 5 | 7 | 6 | 5 |  |  |  |
| 613723 | T:209 C:7 | T:78 C:5 | 7 | 5.71 | 8 | 0.26 | 4 | 8 | 2 | 5 | 8 | 8 | 5 |  |  |  |
| 1586579 | T:79 C:6 | T:37 C:5 | 6 | 5.66 | 12 | 0.27 | 12 | 5 | 7 | 3 | 3 | 4 |  |  |  |  |
| 1898466 | A:100 T:3 | A:37 T:5 | 3 | 5.66 | 10 | 0.27 | 3 | 4 | 10 |  |  |  |  |  |  |  |
| 621901 | T:25 C:310 | T:10 C:133 | 25 | 5.64 | 20 | 0.27 | 15 | 3 | 7 | 15 | 4 | 2 | 3 | 3 | 20 | 2 |
| 834646 | A:5 G:382 | A:5 G:282 | 5 | 5.6 | 13 | 0.27 | 13 | 5 | 4 | 2 | 4 |  |  |  |  |  |
| 170115 | T:5 C:448 | T:5 C:299 | 5 | 5.6 | 13 | 0.27 | 13 | 5 | 4 | 2 | 4 |  |  |  |  |  |
| 834762 | T:40 C:215 | T:10 C:51 | 40 | 5.575 | 20 | 0.27 | 5 | 5 | 6 | 6 | 9 | 1 | 10 | 5 | 8 | 4 |
| 2319109 | A:498 G:7 | A:176 G:5 | 7 | 5.57 | 10 | 0.27 | 5 | 6 | 3 | 10 | 7 | 4 | 4 |  |  |  |
| 2632893 | A:7 G:115 | A:5 G:37 | 7 | 5.57 | 18 | 0.27 | 15 | 3 | 3 | 18 | 2 | 9 | 4 |  |  |  |
| 1429044 | T:498 C:7 | T:176 C:5 | 7 | 5.57 | 10 | 0.27 | 5 | 6 | 3 | 10 | 7 | 4 | 4 |  |  |  |
| 1802639 | T:498 C:7 | T:176 C:5 | 7 | 5.57 | 10 | 0.27 | 5 | 6 | 3 | 10 | 7 | 4 | 4 |  |  |  |
